# Phytochemical Composition and Functional Properties of Brassicaceae Microgreens: Impact of In Vitro Digestion

**DOI:** 10.3390/ijms252111831

**Published:** 2024-11-04

**Authors:** Ivana Šola, Valerija Vujčić Bok, Maja Popović, Sanja Gagić

**Affiliations:** 1 Department of Biology, Faculty of Science, University of Zagreb, Horvatovac 102a, 10000 Zagreb, Croatia; vvujcicbok@pharma.hr (V.V.B.); mpopovic@sumfak.unizg.hr (M.P.); gagic.sanja@gmail.com (S.G.); 2 Division for Pharmaceutical Botany, Faculty of Pharmacy and Biochemistry, University of Zagreb, Ante Kovačića 1, 10000 Zagreb, Croatia

**Keywords:** α-amylase, α-glucosidase, antiglycation, *Brassica*, flavonoids, glucosinolates, *Lepidium*, phenolic acids, *Raphanus*, sinapic acid

## Abstract

The aim of this study was to compare the concentration of phenolic compounds, glucosinolates, proteins, sugars and vitamin C between kohlrabi (*Brassica oleracea* var. *acephala gongylodes*), Savoy cabbage (*B. oleracea sabauda*), Brussels sprouts (*B. oleracea gemmifera*), cauliflower (*B. oleracea botrytis*), radish (*Raphanus sativus*) and garden cress (*Lepidium sativum*) microgreens for their antioxidant and hypoglycemic potential. In addition, we applied an in vitro-simulated system of human digestion in order to track the bioaccessibility of the selected phenolic representatives, and the stability of the microgreens’ antioxidant and hypoglycemic potential in terms of α-amylase and α-glucosidase inhibition after each digestion phase. Using spectrophotometric and RP-HPLC methods with statistical analyses, we found that garden cress had the lowest soluble sugar content, while Savoy cabbage and Brussels sprouts had the highest glucosinolate levels (76.21 ± 4.17 mg SinE/g dm and 77.73 ± 3.33 mg SinE/g dm, respectively). Brussels sprouts were the most effective at inhibiting protein glycation (37.98 ± 2.30% inhibition). A very high positive correlation (*r* = 0.830) between antiglycation potential and conjugated sinapic acid was recorded. For the first time, the antidiabetic potential of microgreens after in vitro digestion was studied. Kohlrabi microgreens best inhibited α-amylase in both initial and intestinal digestion (60.51 ± 3.65% inhibition and 62.96 ± 3.39% inhibition, respectively), and also showed the strongest inhibition of α-glucosidase post-digestion (19.22 ± 0.08% inhibition). Brussels sprouts, cauliflower, and radish had less stable α-glucosidase than α-amylase inhibitors during digestion. Kohlrabi, Savoy cabbage, and garden cress retained inhibition of both enzymes after digestion. Kohlrabi antioxidant capacity remained unchanged after digestion. The greatest variability was seen in the original samples, while the intestinal phase resulted in the most convergence, indicating that digestion reduced differences between the samples. In conclusion, this study highlights the potential of various microgreens as sources of bioactive compounds with antidiabetic and antiglycation properties. Notably, kohlrabi microgreens demonstrated significant enzyme inhibition after digestion, suggesting their promise in managing carbohydrate metabolism and supporting metabolic health.

## 1. Introduction

Germination is an inexpensive, simple, fast and effective way to accumulate bioactive compounds in vegetables. Namely, the concentration of different bioactive compounds increases during seed germination resulting in sprouts with higher biopotential than the seeds from which they originate [1]. On the other side, once germinated, the qualitative and quantitative content of bioactive compounds, as well as the bioactivity of extracts, varies depending on the stage of plant development [2,3]. Since microgreens represent young tissue that is biochemically very active, in many cases, they are richer in bioactive compounds than mature individuals [4]. For example, Chinese cabbage sprouts contain more salicylic acid and vitamin C than mature plants [3]. Sprouts of some cereals contain higher concentrations of bioactive compounds and show more significant positive biological effects on the human body than non-germinated seeds [5]. In Asian countries, the consumption of fresh microgreens is common, while in European countries it is somewhat rarer, but awareness of fresh, functional foods is growing, so the use of microgreens is becoming more common on all continents. The most common microgreens in the diet so far are those from the families Brassicaceae, Asteraceae, Chenopodiaceae, Lamiaceae, Apiaceae, Amarillydaceae, Amaranthaceae and Cucurbitaceae [6]. Since very little information is available on the bioaccessibility of compounds and the antioxidant potential of microgreens after digestion [7], and, to the best of our knowledge, there are no data on their antidiabetic potential upon digestion at all, it is necessary to analyze the same to make the knowledge on their biopotential more complete.

Varieties from the genus *Brassica* (such as broccoli, kale, cauliflower, and Chinese cabbage) belong to the group of ten of the most economically important vegetables [8]. The consumption of cruciferous (Brassicaceae) mature plants is common, and now, due to the wealth of phytochemicals, the consumption of their microgreens is also becoming more frequent [9]. This type of microgreen is especially rich in glucosinolates, and their derivatives isothiocyanates and phenolics [10]. Different molecular pathways along which cruciferous microgreens act have been revealed so far, such as the inhibition of carcinogens binding to DNA, DNA repair, reduction of cell proliferation and angiogenesis, ability to enhance the antioxidant machinery of cells [10]. Along with the genus *Brassica*, both microgreens and mature plants of genera *Raphanus* and *Lepidium* are also very represented in everyday human diets [11,12]. A plethora of health benefits of cruciferous microgreens under different pathophysiological conditions, such as diabetes, hypertension, inflammation and oxidative stress, hepatic and renal toxicity, skin disorders, asthma, etc., have also been revealed [10]. The environmental conditions under which the microgreens are grown can significantly change their nutritional value [13,14,15,16], therefore, may should be considered as a way of producing microgreens with added value. The phytochemical profile and bioactivity of brassicaceous sprouts may even be improved via interspecies transfer of metabolites [17,18].

Plant extracts with amylase and glucosidase inhibitory properties are being investigated for their potential role in managing blood glucose levels, particularly in the context of diabetes [19,20,21,22]. Non-enzymatic protein glycation and the accumulation of advanced glycation products (AGEs) is a pathogenic mechanism related to diabetes, among many other diseases [23]. Finding AGE inhibitors from natural sources is a promising strategy for the prevention of these ailments. So far, AGE formation inhibition properties have been shown for different bioactive compounds and extracts of mature plants [23,24], and there are also clear data on the antiglycation potential of microgreens [25,26]. Among the plant bioactive compounds, flavonoids have emerged as very promising in the inhibition of α-amylase and α-glucosidase activity [27] and the reduction of AGEs [28]. Saponins and triterpenoids such as oleanolic and ursolic acid inhibit both α-amylase and α-glucosidase, impacting carbohydrate breakdown, and also reducing protein glycation through various mechanisms, including antioxidant activity [29].

Bioaccessibility is the proportion of a substance that is released from its matrix in food during digestion and becomes available for absorption [30,31,32]. It depends on different factors, such as the food matrix, that is the structure of the food and the way the substance is bound within it, and digestibility, that is, the ease with which the substance can be released during digestion. Interactions with other food components also determine the bioaccessibility, some compounds may form complexes with other substances in the food, affecting their release. Due to their tender and young nature, microgreens may have higher bioaccessibility of certain nutrients. The nutrient content is generally more concentrated and may be more easily released during digestion.

With that in mind, the aim of this study was to compare the concentrations of bioactive compounds of kohlrabi (*Brassica oleracea* var. *acephala gongylodes*), kale (*B. oleracea sabauda*), Brussels sprouts (*B. oleracea gemmifera*), cauliflower (*B. oleracea botrytis*), radish (*Raphanus sativus*) and garden cress (*Lepidium sativum*), and their antioxidant and hypoglycemic potential before and after in vitro-simulated human digestion in order to single out a variety of microgreens of higher potential for each of the parameters. For that purpose, using a combination of spectrometric, chromatographic and chemometric analyses, we measured the content of different types of bioactive compounds, antioxidant capacity, antiglycation potential, and antidiabetic potential, and analyzed the effect of in vitro human digestion on phytochemical and functional properties, and statistically revealed the relations between the samples, as well as between the samples and the measured parameters.

The results revealed that the microgreens with the highest amount of glucosinolates were Savoy cabbage and Brussels sprouts, while garden cress had the lowest amount of soluble sugars. The most potent inhibition of protein glycation was Brussels sprouts. The most significant differences in phytochemicals between the original samples were found in the conjugated forms of quercetin and kaempferol. The bioactivity parameter that varied the most across the original samples was the ability to inhibit protein glycation. A very high positive correlation (*r* = 0.830) between antiglycation potential and conjugated sinapic acid was recorded. All pre-digestion microgreen extracts showed antioxidant potential similar to or even higher than the Trolox standard. Kohlrabi antioxidant capacity was not changed after any phase of digestion. Extracts of all microgreens before digestion showed the same or higher degree of α-amylase inhibition than the standard antidiabetic drug acarbose. For the first time, microgreens’ antidiabetic potential after in vitro gastrointestinal digestion was analyzed. Kohlrabi microgreens showed the highest potential to inhibit the activity of α-amylase both in the initial phase of digestion and after intestinal digestion. The activity of α-glucosidase after intestinal digestion was also best inhibited by kohlrabi. Kohlrabi, Savoy cabbage, and garden cress microgreens retained their ability to inhibit α-amylase and α-glucosidase after digestion. The decreasing trend in Euclidean distance, indicating that the samples become more similar during digestion, suggests a homogenization of the measured parameters, that is phytochemicals’ concentration, antioxidant capacity, and antidiabetic potential, as digestion progresses. Overall, this trend implies that although the raw samples differ in their phytochemical profiles and bioactivities, digestion processes may diminish these variations, producing a more uniform response in terms of antioxidant and antidiabetic activities.

## 2. Results and Discussion

An increasing number of review papers highlight the growing recognition of microgreens and their importance for health [33,34,35,36,37,38,39]. Microgreens are young, edible plants that are often more nutrient-dense than their fully mature versions. Their ease of cultivation and fast growth cycle make them accessible for home growers and urban farming, offering a sustainable and fresh food source. With their high nutritional content and eco-friendly nature, microgreens play an important role in promoting human health and addressing food security challenges in modern life.

### 2.1. Amount of Different Groups of Phenolic Compounds in Brassicaceae Microgreens

Kohlrabi microgreens had the highest amount of total phenolics (TP) (23.30 ± 2.44 mg GAE/g dm) and total tannins (TT) (3.96 ± 0.29 mg TAE/g dm) (Table 1). Total hydroxycinnamic acids (THCA) and flavonols were the most represented in Savoy cabbage microgreens with 30.54 ± 1.83 mg CinE/g dm and 63.42 ± 3.52 mg QE/g dm, respectively. The greatest difference between the samples containing the highest and the lowest amount of a certain group of polyphenolics we detected for TT. Namely, the microgreens of kohlrabi had a 70% higher amount of TT compared to the microgreens of Brussels sprouts. Since both kohlrabi and Brussels sprouts belong to the same genus (*Brassica*), while radish and garden cress are genetically more distant, this result shows that gender affiliation does not correlate with the amount of TT. The content of TP and THCA was the least different between the samples, kohlrabi had 35% higher content of TP than radish, and Savoy cabbage had 35% higher content of THCA than Brussels sprouts.

### 2.2. Amount of Total Glucosinolates, Proteins and Soluble Sugars in Brassicaceae Microgreens

**Figure 1:**
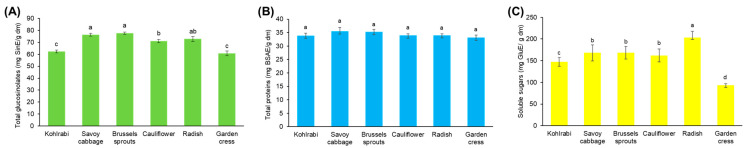
Amount of (**A**) total glucosinolates, (**B**) proteins and (**C**) soluble sugars in Brassicaceae microgreens. Values represent mean ± standard deviation of three biological and three technical replicates. Different letters indicate a significant difference among the samples (ANOVA, Duncan test, *p* ≤ 0.05). SinE = sinigrin equivalent, BSAE = bovine serum albumin equivalent, GluE = glucose equivalent, dm = dry mass.

Glucosinolates in plants exhibit fungicidal, bactericidal, nematocidal, and allelopathic effects [40]. Beyond their anticancer benefits, consuming glucosinolates promotes cardiovascular health, metabolic function, cognitive abilities, and musculoskeletal health [41]. While the accumulation of glucosinolates in plants enhances their resistance to environmental stresses, their protective and biological effects in humans are mainly mediated by their metabolic byproducts, especially isothiocyanates [42]. For example, recent research has found that isothiocyanate sulforaphane, when used alongside anti-cancer therapies such as chemotherapy, boosts the sensitivity of cancer cells and reduces their harmful side effects [43,44]. The highest amount of total glucosinolates we recorded were in Savoy cabbage and Brussels sprout microgreens, 76.21 ± 4.17 mg SinE/g dm and 77.73 ± 3.33 mg SinE/g dm, respectively (Figure 1A). The lowest amounts were recorded in kohlrabi and garden cress microgreens. The number of total proteins was not significantly different between the tested types of microgreens, and it was in the range of 33.14 ± 0.83 mg BSAE/g dm in garden cress and 35.45 ± 1.45 mg BSAE/g dm in Savoy cabbage. On the other hand, soluble sugars were differently represented with radish microgreens having the highest amount (202.98 ± 4.05 mg GluE/g dm) and garden cress the lowest amount (93.05 ± 4.71 mg GluE/g dm). It is interesting to note that all microgreens of the genus *Brassica* were between radish (*Raphanus*) and garden cress (*Lepidium*) in terms of soluble sugar amounts. Moreover, the content of soluble sugars did not significantly differ between Savoy cabbage, Brussels sprouts and cauliflower, but it was significantly higher than in kohlrabi microgreens.

The total protein content among microgreens in the Brassicaceae family, including genera *Brassica*, *Raphanus* and *Lepidium*, is typically less variable compared to soluble sugars [45]. This might be due to several factors tied to the plant family, developmental stage, and metabolic regulation. The Brassicaceae family shares a common evolutionary history, resulting in similar metabolic pathways for protein synthesis. Since proteins are fundamental for cellular structure and function, the general protein composition tends to be relatively stable across species within a family, especially at the microgreen stage where all plants are young and in the early stages of development. Basic protein requirements for cellular growth are conserved, so the total protein content in microgreens from different genera of Brassicaceae may not show drastic variation [46,47]. Proteins are crucial for all stages of growth, particularly in young plants like microgreens. Microgreens are harvested during their early growth stages, where cellular machinery, enzymes, and structural proteins are necessary for rapid development. Because these processes are universally important across species, the overall protein content is relatively constant among Brassicaceae microgreens. Soluble sugars, on the other hand, are products of photosynthesis and energy storage. They are highly influenced by environmental conditions such as light [48,49,50], temperature [51,52,53,54,55], and nutrient availability [56,57,58], which can vary widely even among closely related species. Sugars also vary based on metabolic demands, which can differ more between species due to different growth rates or photosynthetic capacities [59,60]. Soluble sugars like glucose, fructose, and sucrose accumulate during photosynthesis [61]. Differences in photosynthetic efficiency, leaf size, and overall energy metabolism across genera like *Brassica, Raphanus*, and *Lepidium* can lead to more variation in soluble sugar content. Different plants have evolved to store and utilize energy in diverse ways. Some microgreens may allocate more sugars to growth and energy reserves, leading to variations in soluble sugar levels. For instance, radish microgreens (*Raphanus*) might accumulate more sugars compared to others due to faster growth rates, resulting in greater energy demands [39]. Although all plants require proteins for structural components and enzymes, the allocation of metabolic resources to sugars can differ based on species-specific growth strategies. Some microgreens may prioritize rapid energy accumulation for fast growth, while others may invest in different metabolic pathways. Since proteins are more universally necessary for plant structure and enzyme activity, they tend to show less variability than sugars, which are more influenced by external factors and internal metabolic priorities [62].

### 2.3. Concentration of Vitamin C and Individual Phenolic Compounds in Brassicaceae Microgreens

The chromatograms of Brassicaceae microgreens are shown in Figures S1 and S2. The highest concentration of free *L*-ascorbic acid was recorded in radish microgreens (1956.43 ± 71.47 mg/kg dm) (Table 2). On the other hand, garden cress had the highest concentration of derivatized *L*-ascorbic acid (1218.41 ± 45.95 mg/kg dm). Brussels sprouts contained the highest concentration of derivatized ferulic acid (104.59 ± 0.39 mg/kg dm), while Savoy cabbage showed the highest concentration of derivatized sinapic acid (1668.71 ± 29.46). What caught our attention was the fact that in the analyzed microgreens, derivatized sinapic acid was present in around 10 times higher concentration than free form. Also, in each of the varieties, sinapic acid was present in a higher concentration than ferulic acid. We assume one of the reasons might be evolutionary adaptation. Namely, sinapic acid and its derivatives, such as sinapoyl esters, play a crucial role in plant defense against herbivores, pathogens, and environmental stressors like UV radiation [63,64]. Sinapic acid derivatives are more efficient than ferulic acid in UV absorption, which provides protection to plant tissues, particularly in leafy vegetables like microgreens that we used [65,66]. This enhanced protection could explain the higher concentration of sinapic acid in these plants.

Free quercetin was recorded in cauliflower and radish microgreens only, and radish contained a significantly higher concentration (302.98 ± 7.64 mg/kg dm). On the other hand, derivatized quercetin was recorded in all the samples, with the concentration range from 101.25 ± 1.28 mg/kg dm in radish up to 638.66 ± 16.19 mg/kg dm in cauliflower microgreens. Therefore, in all the tested samples, quercetin was dominantly present in derivatized forms, except for radish where free form was represented with three times higher concentration than derivatized. Kaempferol was detected in derivatized forms only, and the highest concentration was present in radish (840.45 ± 34.08 mg/kg dm).

In general, all the identified phenolic compounds were present in higher concentrations in the derivatized form(s) than in the free form(s), except ferulic acid in kohlrabi and quercetin in radish. The fact that derivatized forms of phenolics are dominant over free might be due to protection from degradation, detoxification, storage and/or transport. Regarding the exceptions that we detected, we hypothesize that a higher concentration of free than derivatized ferulic acid in kohlrabi microgreens could be due to the lower activity of the feruloyl esterase enzyme. Also, higher concentrations in free rather than derivatized quercetin in radish might suggest a lower activity of glycosyltransferases in these microgreens.

### 2.4. Comparison of Antioxidant Capacity of Brassicaceae Microgreens Extracts

According to the ABTS method, the highest potential to inhibit oxidation was recorded for cauliflower extracts—81.95 ± 1.33% of inhibition (Table 3). FRAP assay revealed kohlrabi extract as the most potent in antioxidant activity (94.48 ± 0.20% of inhibition). The DPPH results showed that all the samples from the *Brassica* genus were significantly better in antioxidant capacity than radish and garden cress. Moreover, this assay clearly separated each of the genera on its own in the following order *Brassica* > *Raphanus* > *Lepidium*. The difference in the results between the methods used was expected since DPPH is more suitable for lipophilic antioxidants, FRAP for hydrophylic antioxidants, and ABTS can reliably detect both [67,68]. Based on the results, we conclude that microgreens from the genus *Brassica* contain more lipophilic antioxidants than radish and garden cress.

### 2.5. Potential of Brassicaceae Microgreens to Inhibit Protein Glycation

**Figure 2:**
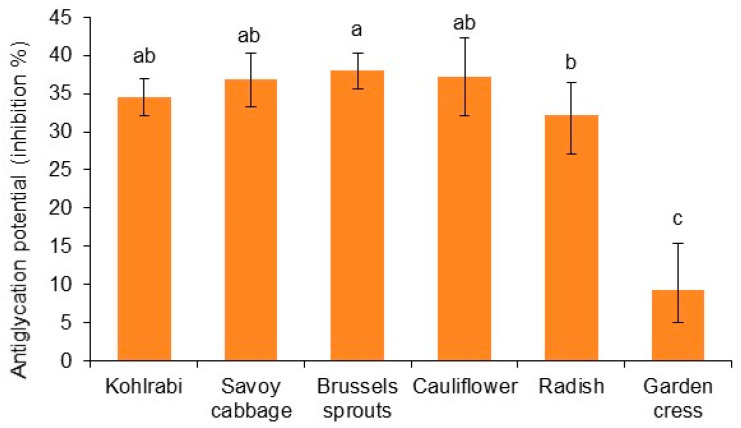
Potential to inhibit protein glycation expressed in percentage of inhibition (%). Different letters indicate a significant difference among the values (ANOVA, Duncan test, *p* ≤ 0.05).

Advanced glycation end-products (AGEs) are harmful compounds formed when sugars, particularly glucose, react non-enzymatically with proteins. Glycation alters the structure and function of proteins, rendering them less efficient or even dysfunctional. AGEs contribute to a variety of chronic diseases and the aging process. Glycation inhibitors and AGE inhibitors are being explored for their potential to slow the progression of diseases linked to glycation, particularly diabetes and hyperglycemia, aging-related disorders like cataracts, neurodegenerative disorders, cardiovascular diseases and arthritis [69,70]. Glycation inhibitors and AGE inhibitors are being explored for their potential to slow the progression of diseases linked to glycation [71]. In our work, the highest potential to inhibit protein glycation we recorded was for Brussels sprouts, 37.98 ± 2.30% inhibition (Figure 2). Radish microgreens had significantly lower potential, and the lowest we detected was for garden cress. This perfectly correlates with the DPPH results (Table 3), so we assume that lipophilic antioxidants were crucial in the reduction of oxidative stress that accelerates the glycation process and the formation of AGEs. Regarding the lowest activity of garden cress, we recorded a very high positive correlation (*r* = 0.830) between antiglycation potential and conjugated sinapic acid (Table 2). The range of correlation coefficient values and the corresponding levels of correlation were interpreted according to Evans [72]. A similar link was also noticed by Navarro et al. [73] working on rapeseed by-products, and by Thilavech et al. [74] studying *Brassica* vegetables, so this should definitely be investigated in more detail.

### 2.6. Amount of Brassicaceae Microgreens Phenolics After In Vitro Human Digestion

The amount of total phenolics (TP) in Brassicaceae microgreens after in vitro simulation of the initial salivary and gastric phases did not differ significantly between the samples (Table 4). However, after intestinal digestion, the highest amount of TP remained in Savoy cabbage (15.75 ± 1.35 mg GAE/g dm). On the other hand, total flavonoids (TF) varied much more after each stage of digestion. After intestinal digestion, TF was the highest in cauliflower (24.70 ± 1.14 mg QE/g dm). The highest concentration of ferulic acid after intestinal digestion we recorded was in Brussels sprouts, 148.49 ± 1.99 mg/kg dm, while the highest concentration of sinapic acid after intestinal digestion we detected was in Savoy cabbage (2197.03 ± 43.06 mg/kg dm). Cauliflower had the highest concentration of quercetin (466.60 ± 7.85 mg/kg dm), and radish had the highest concentration of kaempferol (454.12 ± 19.25 mg/kg dm). Total identified phenolic acids (TIPA), as well as total identified phenolics (TIP) after intestinal digestion were most present in Savoy cabbage (2.28 ± 0.04 g/kg dm and 2.70 ± 0.05 g/kg dm, respectively), while total identified flavonoids (TIF) were prevalent in cauliflower (0.79 ± 0.01 g/kg dm).

Regarding the stability after digestion, TP of Brussels sprouts, cauliflower and garden cress after the intestinal phase were significantly reduced, while the amount of those in kohlrabi, Savoy cabbage and radish was not significantly affected. The bioaccessibility of total flavonoids after intestinal digestion was significantly increased from each of the microgreens, except kohlrabi where it was not changed. We assume this might be due to the enzymatic decomposition of flavonoids bound to fibers or complex structures in plant cells. This process liberates flavonoids, increasing their bioaccessibility compared to raw or undigested microgreens. A similar effect was reported by Bashmil et al. [75] with different samples of green bananas. The bioaccessibility of TF after the intestinal phase of digestion was increased, compared to the initial phase, from each of the microgreens except kohlrabi, where it was not significantly changed after any of the digestion phases. *L*-Ascorbic acid after the intestinal phase of digestion could not be measured due to interfering compounds which are probably similar in structure to *L*-ascorbic acid and which interfered with the measurement, giving unusually high concentrations (Table 5). However, up to the gastric phase, the data could be interpreted with sufficient confidence. Compared to the initial stage, after the gastric phase, the bioaccessibility of *L*-ascorbic acid from kohlrabi, Savoy cabbage and radish was increased, while from garden cress it was decreased. *L*-ascorbic acid bioaccessibility from Brussels sprouts and cauliflower was not affected by the digestion process. The bioaccessibility of ferulic acid from all the *Brassica* samples increased after the intestinal phase, from radish it decreased, while from garden cress it was not changed. Sinapic acid was the only identified compound whose bioaccessibility from any of the samples after the intestinal phase was not changed, compared to the initial phase. Quercetin was more bioaccessible from Savoy cabbage and Brussels sprouts, and less bioaccessible from kohlrabi, cauliflower and garden cress. Intestinal digeston did not affect the bioaccessibility of quercetin from radish. Intestinal digestion negatively affected the bioaccessibility of kaempferol from kohlrabi, cauliflower, radish and garden cress, while it increased it from Savoy cabbage. The total identified phenolic acid bioaccessibility from any of the microgreen samples after intestinal digestion was not affected, while TIF accessibility was either reduced (kohlrabi, cauliflower, radish and garden cress) or increased (Savoy cabbage and Brussels sprouts).

### 2.7. Comparison of Antioxidant Capacity of Brassicaceae Microgreens Extracts After In Vitro Human Digestion

The highest antioxidant capacity measured by the ABTS, DPPH and FRAP methods after the intestinal phase of digestion was the kohlrabi microgreens (21.66 ± 3.34, 25.92 ± 2.61, 72.48 ± 0.25% of inhibition, respectively) (Table 6). The fiber content in kohlrabi could aid in a slower release of nutrients during digestion, promoting prolonged antioxidant effects. Kohlrabi might contain a unique combination of antioxidants, vitamins, and minerals that work synergistically, resulting in higher total antioxidant activity even after digestion. Even if the original phytochemicals do not correlate with antioxidant activity, their breakdown products formed during digestion could have strong antioxidant properties. For instance, glucosinolates are broken down into isothiocyanates and indoles during digestion, which may have stronger antioxidant effects than the parent compounds. Similarly, phenolic compounds can be metabolized by gut microbiota into smaller molecules that are highly bioactive and may exert stronger antioxidant effects. Dietary fibers present in kohlrabi could indirectly influence antioxidant activity. Namely, the fibers can bind to polyphenols and other compounds, protecting them from degradation in the stomach and releasing them gradually in the intestines [76]. This slow release could lead to enhanced antioxidant activity post-digestion, even if the raw content of the specific phytochemicals does not correlate. There might be other antioxidant compounds in kohlrabi that have not been fully identified or quantified. For instance, minor or non-traditional antioxidants (like carotenoids, tocopherols or proteins with antioxidant activity) could contribute significantly to its antioxidant capacity. When we monitored the antioxidant capacity after digestion, we detected that for each microgreen sample, except for kohlrabi, the potential to inhibit ABTS^+^ was significantly reduced after the intestinal phase of digestion. Kohlrabi’s antioxidant capacity was not changed after any phase of digestion. This is another point supporting our hypothesis that kohlrabi could contain a unique combination of antioxidants, vitamins, and minerals that work synergistically. The potential of kohlrabi, Brussels sprouts and radish extracts to inhibit DPPH˙radicals after the final intestinal phase was not significantly changed. On the other hand, the potential of Savoy cabbage, cauliflower and garden cress to inhibit DPPH˙radicals was reduced after the final digestion phase. Antioxidant capacity measured using the FRAP method showed a reduction in potential after intestinal digestion for each of the microgreen types. Since FRAP detects hydrophilic antioxidants, we assume that hydrophilic antioxidant compounds were negatively affected by the digestion process. Among the samples, garden cress antioxidant capacity, measured by the ABTS and FRAP methods, was most negatively affected by the digestion process.

### 2.8. Comparison of Brassicaceae Microgreens’ Potential to Inhibit Enzymes α-Amylase and α-Glucosidase

Both α-amylase and α-glucosidase are key enzymes involved in carbohydrate metabolism, breaking down complex carbohydrates into simpler sugars like glucose, which are then absorbed into the bloodstream. In individuals with diabetes, excessive glucose absorption leads to hyperglycemia, which, over time, can cause serious complications such as cardiovascular disease, neuropathy, and retinopathy. By inhibiting these enzymes, plant-derived compounds can slow the breakdown of carbohydrates, resulting in a more gradual release of glucose into the blood [77,78,79,80]. This modulation of postprandial (after eating) blood glucose levels is vital for effective diabetes management. Exploring the natural potential of plants to inhibit the activity of these enzymes offers several advantages over synthetic inhibitors. Namely, plant-based compounds tend to have fewer side effects and are generally well-tolerated by the body. In our study, first what we detected is that microgreens showed a higher potential to inhibit α-amylase than α-glucosidase (Table 7). Amylase and glucosidase are structurally different enzymes with distinct substrate specificities. Amylase hydrolyzes large polysaccharides like starch into smaller sugars (maltose, glucose), while glucosidase hydrolyzes disaccharides into glucose. Plant compounds, especially polyphenols and flavonoids, may have a greater affinity for binding to amylase due to its larger and more open active site compared to glucosidase, which typically works on smaller, more specific substrates [80]. The polyphenols, flavonoids, and tannins present in plant extracts are known to bind to proteins through hydrogen bonding and hydrophobic interactions. Amylase, with its larger size and more accessible active site, might provide more binding opportunities for these compounds compared to glucosidase, resulting in more effective inhibition of amylase than glucosidase [81]. Amylase is more sensitive to inhibitors from plant extracts than glucosidase. Studies have shown that plant polyphenols like quercetin, rutin, and other flavonoids more strongly inhibit amylase than glucosidase, possibly due to differences in the enzymes’ susceptibility to competitive or non-competitive inhibition [81]. Another fact that is important to take into account is that amylase is active in the slightly alkaline environment of the small intestine (pH 6.7–7.0), while glucosidase functions optimally in a broader range of pH values. In vitro digestion conditions (e.g., pH, enzyme concentration) might be more favorable for the inhibition of amylase than glucosidase [82].

Kohlrabi microgreens showed the highest potential to inhibit the activity of α-amylase both in the initial phase of digestion and after intestinal digestion (60.51 ± 3.65 and 62.96 ± 3.39% of inhibition, respectively). The least potential after intestinal digestion was recorded for Brussels sprouts (23.56 ± 4.57% of inhibition). The activity of α-glucosidase after intestinal digestion was also best inhibited by kohlrabi, with 19.22 ± 0.08% of inhibition. Similar to α-amylase, Brussels sprouts were least effective in the inhibition of α-glucosidase, with 2.88 ± 1.82% of inhibition. Interestingly to note is that against α-glucosidase, the most effective in the initial phase were radish microgreens (18.70 ± 0.62% inhibition), however, after the intestinal phase of digestion, this potential was significantly reduced and not the highest among the analyzed samples. A considerable inhibition of α-glucosidase by radish microgreens had already been detected by Wojdyło et al. [4]. When we looked at the stability of microgreens’ inhibitory potential against α-amylase after in vitro digestion, we noticed that the inhibition was significantly reduced after the intestinal phase only by Brussels sprouts (reduction of 48% compared to the initial phase), while all the other samples, except kohlrabi and radish, showed an increase. We hypothesize this might be due to the pH-dependent inhibitory effects of phenolic compounds on α-amylase. Namely, the inhibitory potential of some bioactive compounds is higher under the neutral pH conditions typical of the intestinal phase [83,84]. Additionally, it is known that bile salts and digestive products facilitate the activity of some plant polyphenols, enhancing their ability to interact with enzymes in the intestinal phase [77,85]. On the other hand, the stability of microgreens’ inhibitory potential against α-glucosidase after in vitro digestion was significantly decreased after the intestinal phase by Brussels sprouts (reduction of 72%), cauliflower (reduction of 54%) and radish (reduction of 67%). This suggests that the phytochemicals of Brussels sprouts, cauliflower and radish that are responsible for α-glucosidase inhibition are more susceptible to digestion than their phytochemicals responsible for α-amylase inhibition. Overall, the microgreens whose potential to inhibit both α-amylase and α-glucosidase were not significantly reduced after digestion were kohlrabi, Savoy cabbage and garden cress.

### 2.9. Chemometric Data Analysis

#### 2.9.1. Principal Component Analysis

**Figure 3:**
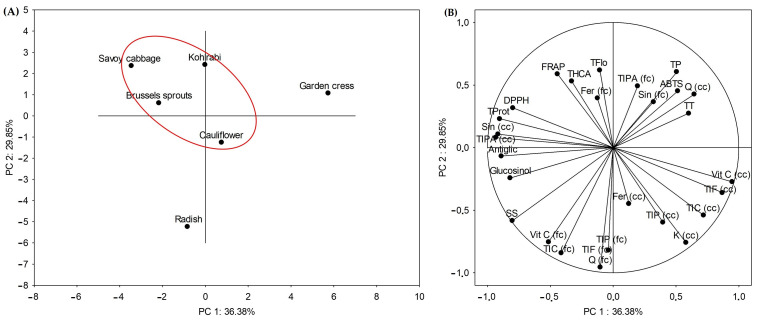
Diagram (biplot) of Principal Component Analysis (PCA) of undigested microgreens based on their total and individual bioactive compounds, antiglycation and antioxidant potential. (**A**) Grouping of samples, (**B**) grouping of analyzed parameters. TP = total phenolics, TFlo = total flavonols, TT = total tannins, THCA = total hydroxycinnamic acids, SS = soluble sugars, Glucosinol = total glucosinolates, ABTS = antioxidant capacity measured by the method ABTS, FRAP = antioxidant capacity measured by the FRAP method, DPPH = antioxidant capacity measured by the DPPH method, Antiglic = antiglication potential, Vit C = vitamin C, Fer = ferulic acid, Sin = sinapic acid, Q = quercetin, K = kaempferol, TIPA = total identified phenolic acids, TIF = total identified flavonoids, TIP = total identified phenolics, TIC = total identified compounds, fc = free compound, cc = conjugated compound.

Principal component analysis (PCA) helps to reduce the number of variables (dimensions) into a new set of variables called principal components, which represent linear combinations of the original data. The components in PCA explain portions of the variance in the data, which means that two points close in a PCA plot might not belong to the same cluster but rather share similarities along certain axes. Figure 3 shows the separation of undigested microgreens based on their total and individual bioactive compounds, antioxidants and antiglycation potential. It is evident that the samples belonging to the *Brassica* genus share similarities and are separated from radish and garden cress, and this was expected. Namely, radish and garden cress belong to the genera *Raphanus* and *Lepidium*, respectively. Another thing that we detected is that cauliflower shared a higher level of similarities with radish and garden cress, than the other *Brassica* microgreens. One of the possible explanations for this might be convergent adaptation and/or selective breeding.

**Figure 4:**
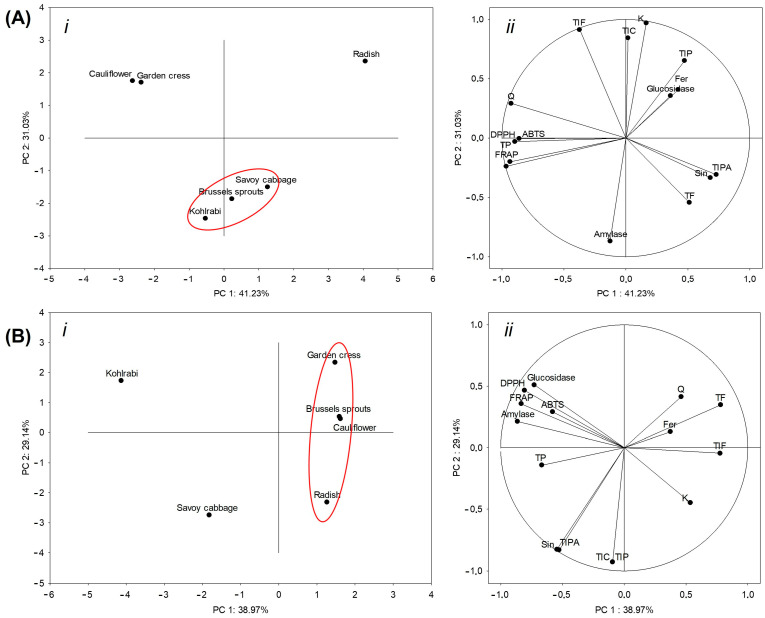
Diagram (biplot) of Principal Component Analysis (PCA) of microgreens after (**A**) initial and (**B**) intestinal phase of digestion based on their total and individual bioactive compounds, antioxidant potential, and ability to inhibit enzymes α-amylase and α-glucosidase. (*i*) Grouping of samples, (*ii*) grouping of analyzed parameters. TP = total phenolics, TF = total flavonoids, ABTS = antioxidant capacity measured by the method ABTS, FRAP = antioxidant capacity measured by the FRAP method, DPPH = antioxidant capacity measured by the DPPH method, Fer = ferulic acid, Sin = sinapic acid, Q = quercetin, K = kaempferol, TIPA = total identified phenolic acids, TIF = total identified flavonoids, TIP = total identified phenolics, TIC = total identified compounds.

Separations of microgreens after the initial (Figure 4A) and the intestinal (Figure 4B) phase of digestion reveal changes in the mutual grouping of samples. Namely, after the initial phase, kohlrabi, Brussels sprouts and Savoy cabbage formed one separate cluster, cauliflower and garden cress another, and radish was separated from both clusters (Figure 4(A*i*)). Variables that contributed the most to the grouping of kohlrabi, Brussels sprouts and Savoy cabbage were the level of α-amylase inhibition, concentration of sinapic acid, total flavonoids and total identified phenolic acids (Figure 4(A*ii*)). The rate of inhibition of α-glucosidase, the concentration of ferulic acid and the total identified phenolics contributed the most to the separation of radish from the other groups. Following intestinal digestion, the arrangement of the samples in the diagram was altered. Brussels sprouts, cauliflower, garden cress, and radish formed a single cluster, whereas kohlrabi and Savoy cabbage were distinctly separated from both the cluster and each other (Figure 4(A*ii)*). The most similar to each other were Brussels sprouts and cauliflower. Parameters that contributed predominantly to the separation of kohlrabi were the results of the antioxidant assays ABTS, FRAP and DPPH, and the level of inhibition of α-amylase and α-glucosidase. The separation of Savoy cabbage was primarily influenced by the concentration of sinapic acid and the total identified phenolic acids. Notably, after the intestinal digestion phase, the results of the antioxidant assays ABTS, DPPH, and FRAP, along with the inhibition levels of the antidiabetic enzymes α-amylase and α-glucosidase, were concentrated in one-quarter of the diagram, while the other measured parameters were distributed across the remaining three quarters. This suggests that the antioxidant capacity and inhibition of enzymes related to antidiabetic effects are closely linked and behave similarly following intestinal digestion. In contrast, other measured parameters show more variability, indicating distinct responses to digestion. Such clustering of antioxidant and enzyme inhibition results could point to a stronger correlation between these factors in post-digestion conditions.

#### 2.9.2. Hierarchical Clustering

**Figure 5:**
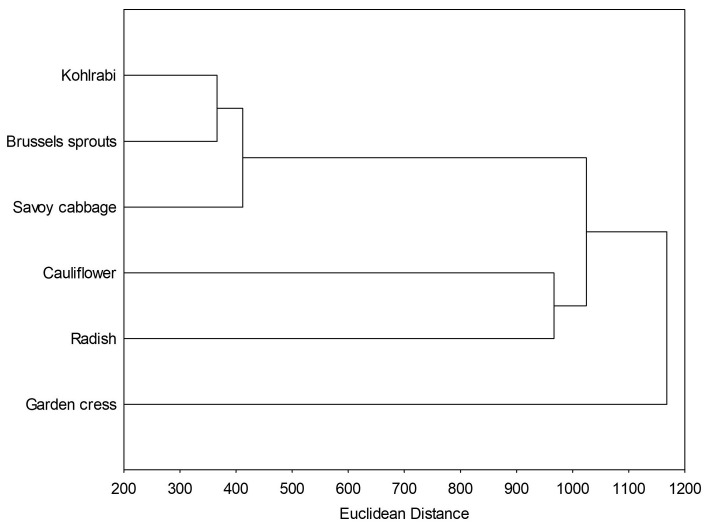
Hierarchical clustering, expressed as Euclidean distance, of undigested microgreens, based on their total and individual bioactive compounds, antiglycation and antioxidant potential.

Hierarchical clustering is an unsupervised machine-learning algorithm used to group similar objects into clusters based on their distance or similarity. It provides a detailed view of relationships between data points, offering insight into data structure through dendrograms. Figure 5 shows the relationships between the microgreens before digestion based on their total and individual bioactive compounds, antioxidant and antiglycation potential. It is evident that kohlrabi, Brussels sprouts and Savoy cabbage formed two close clusters, while cauliflower was more distant and formed the cluster with radish from the genus *Raphanus*. This suggests that the phytochemical traits of cauliflower are not closely linked to its evolutionary and genetic factors. Garden cress was most distant from all the samples.

**Figure 6:**
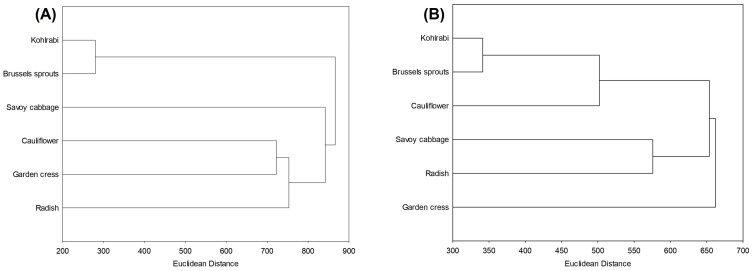
Hierarchical clustering, expressed as Euclidean distance, of (**A**) undigested microgreens based on their total and individual bioactive compounds, antiglycation and antioxidant potential; and (**B**) microgreens after intestinal phase of digestion based on their total and individual bioactive compounds, antioxidant potential, and ability to inhibit enzymes α-amylase and α-glucosidase.

The hierarchical clustering of the samples after the initial and intestinal phases of digestion is shown in Figure 6. The most noticeable trend was that the Euclidean distance between the samples decreased progressively, the highest was between the original samples, 1200 (Figure 5), then after the initial phase was 900 (Figure 6A), and finally after the intestinal phase it was 700 (Figure 6B). The decreasing trend in Euclidean distance, indicating that the samples become more similar during digestion, suggests a homogenization of the measured parameters, that is phytochemicals’ concentration, antioxidant activity, and antidiabetic activity, as digestion progresses. Overall, this trend implies that although the raw samples differ in their phytochemical profiles and bioactivities, digestion processes may diminish these variations, producing a more uniform response in terms of antioxidant and antidiabetic activities. Both before and after digestion, the most similar microgreens, based on the measured phytochemicals, antioxidant and antidiabetic potential, were kohlrabi and Brussels sprouts.

#### 2.9.3. Pearson’s Correlation Coefficients

Pearson’s correlation coefficient measures the strength and direction of the linear relationship between two continuous variables in order to test hypotheses about the relationships between variables. Table 8 shows correlation coefficients between measured variables in undigested microgreens. Antioxidant capacity measured using the ABTS method was, according to Evans [82], very strongly positively (*r* = 0.844) correlated with the concentration of conjugated quercetin. Antiglycation activity was very strongly positively correlated with conjugated sinapic acid (*r* = 0.830) and the total identified conjugated phenolic acids (*r* = 0.845).

Table 9 shows the correlation coefficients between the measured variables in microgreens after intestinal digestion. Inhibition of α-amylase activity was very strongly positively (*r* = 0.919) correlated with antioxidant capacity measured using the FRAP method, and strongly positively (*r* = 0.718) with antioxidant capacity measured using the DPPH method. This could be attributed to the presence of specific phytochemicals that possess both antioxidant and α-amylase inhibitory properties. Inhibition of α-glucosidase was very strongly positively (*r* = 0.843) correlated with antioxidant capacity measured using the DPPH method, and strongly positively (*r* = 0.767 and *r* = 0.739) correlated with antioxidant capacity measured using the ABTS and FRAP methods, respectively. The observed correlations suggest a potential synergistic effect where antioxidant compounds not only provide protective benefits against oxidative damage but also play a crucial role in managing carbohydrate digestion and absorption, thus aiding in the regulation of blood sugar levels. This could lead to investigations into the underlying mechanisms by which antioxidants influence enzyme activity and carbohydrate digestion. The correlation coefficients between amylase/glucosidase inhibition and antioxidant potential were higher than those between amylase/glucosidase inhibition and bioactive compounds. This suggests that antioxidants might play a more significant role in enzyme inhibition than other bioactive compounds. Also, a correlation coefficient between α-amylase and α-glucosidase inhibition was very high (*r* = 0.823), which might indicate that the same compounds or mechanisms are responsible for inhibiting both enzymes. A strong correlation in their inhibition could suggest that compounds that inhibit one enzyme may also effectively manage blood sugar levels by inhibiting the other, thus providing a dual benefit. This encourages further research to identify specific inhibitors that could target both enzymes simultaneously, potentially leading to more effective treatments for conditions like diabetes. The study could support the development of functional foods or supplements designed to enhance antioxidant intake while also targeting carbohydrate metabolism.

## 3. Materials and Methods

### 3.1. Chemicals and Materials

Standards of flavonoids and *L*-ascorbic acid were of HPLC grade and purchased either from Sigma Aldrich (GmbH (Taufkirchen, Germany) or from Extrasynthese (Genay, France). All other chemicals and reagents were supplied by Sigma Aldrich GmbH (Taufkirchen, Germany). Unless otherwise specified, the chemicals and reagents were of analytical grade, and the used water was deionized using a Stakpure ion exchange system. Poroshell 120 SB-C18 non-polar column and a Zorbax Rx-C18 guard column were purchased from Agilent (Santa Clara, CA, USA). The seeds of kohlrabi (*Brassica oleracea* var. *acephala gongylodes*, sort Viola), kale (*B. oleracea sabauda*, sort Re d’inverno), Brussels sprouts (*B. oleracea gemmifera*, sort Bruxelles mezzo nano), cauliflower (*B. oleracea botrytis*, Palla di neve X), radish (*Raphanus sativus*, Cherry belle) and garden cress (*Lepidium sativum*) were purchased from Agromlinar d.o.o. (Zagreb, Croatia). The producer of all the seeds was N. Sgaravatti and C. Sementi S.p.a., Italy, except for garden cress that was produced by Green paradise s.r.l., Italy. Seeds were sterilised with 2.55% Izosan^®^ G (Pliva, Zagreb, Croatia) and germinated in a climate chamber at room temperature (RT) on a wet filter paper in the dark. Upon germination, cultivation was continued at RT under illumination cycle 16 h day/8 h dark. When they reached the two-real-leaf stage, they were collected, frozen under liquid nitrogen, lyophilized using an Alpha 1–2 LSCbasic freeze-dryer (Martin Christ Gefriertrocknungsanlagen GmbH, Osterode am Harz, Germany), pulverized using a pestle and mortar, and then used to prepare extracts. Seedlings were grown in 3 biological replicas and for all the analyses 3 technical replicas were prepared from each of the biological replicas.

### 3.2. Extraction of Phytochemicals

Freeze-dried powder of microgreens was extracted with 70% ethanol for determination of total phenolics, flavonoids, flavonols, tannins, soluble sugars, individual phenolics, *L*-ascorbic acid, antioxidant and antidiabetic capacity. Extracts at the concentration of 30 mg/mL were used in all the methods, except for the soluble sugars where a concentration of 0.6 mg/mL was used. For each method, extracts were prepared as follows: solvent was added to the plant material, shaken by vortex mixer for 1 min followed by 20 rpm rotation for 60 min at RT. The obtained extracts were centrifuged for 5 min at 10,000 rpm and the supernatants stored at −20 °C until further analyses.

### 3.3. In Vitro Digestion

In vitro digestion model was performed as described in Šola et al. (2020b) [17] with slight modification. A volume of 0.75 mL of extract was mixed with the same volume of 20 mM phosphate buffer pH 7.0. To initialize salivary phase of digestion 25 μL of amylase (0.48 mg/mL in 20 mM phosphate buffer pH 7.0) was added and incubated for 5 min at 37 °C in a shaking water bath at 150 rpm. For simulating the stomach digestion a volume of 1 mL of porcine pepsin solution (3 mg/mL in 0.1 M HCl) was added to the salivary phase and acidified with 0.5 M HCl (pH 2.0). Samples were incubated in a shaking water bath for 1 h at 37 °C and 150 rpm. Upper intestinal phase of digestion was mimicked first by adding sodium bicarbonate (1 M NaHCO_3_) to gastric phase to adjust pH to 5.3. After pH adjustment volume of 2.25 mL of pancreatic juices (2.4 mg bile acids/mL, 0.2 mg porcine lipase/mL, 0.4 mg pancreatin/mL in 20 mM phosphate buffer pH 7.0) was added. The final total volume of each intestinal phase sample was brought to 5 mL with 20 mM phosphate buffer (pH 7.0). The final pH was adjusted additionally to 7.0 with 1 M NaOH. Samples were then incubated for 2 h at 37 °C in a shaking water bath at 150 rpm. The final volume of each sample, both before and after digestion, was brought to 5 mL with 20 mM phosphate buffer (pH 7.0). Samples were centrifuged at 11,000 rpm for 10 min at 4 °C and supernatants were stored at −20 °C until spectrophotometric and HPLC analyses.

### 3.4. Spectrophotometric Determination of Phytochemicals and Antioxidant Capacity

Total phenols, flavonoids and flavonols were determined as in Poljuha et al. [86] and Šola et al. [3,17]. Total tannins were determined according to Galvão et al. [87] and soluble sugars were determined as in Dubois et al. [88]. Total glucosinolates were determined according to Mawlong et al. [89], and proteins according to Bradford (1976) [90]. Antioxidant activity of nonhydrolyzed extracts was measured by three assays (ABTS or 2,2′-azino-bis(3-ethylbenzothiazoline-6-sulfonic acid), DPPH or 2,2-diphenyl-1-picrylhydrazyl, and FRAP or ferric ion reducing antioxidant power) as described in Poljuha et al. [86]. Antiglycation assay was performed according to Spínola et al. [91]. All absorbance measurements were performed on a Fluostar Optima microplate reader (BMG Labtech GmbH, Offenburg, Germany).

### 3.5. RP-HPLC Analysis of Phenolics and L-Ascorbic Acid

Phenolic compounds and *L*-ascorbic acid were analyzed before and after acid hydrolysis. Hydrolysis was performed by adding HCl 1.2 M followed by incubation for 2 h at 80 °C and 300 rpm. Separation, identification and quantification of compounds were peformed on an Agilent 1100 Series device with UV/Vis detector (Agilent Technologies, Waldbronn, Germany). The separation was carried out on a Poroshell 120 SB-C18 non-polar column (4.6 × 75 mm, 2.7 μm particle size) using a Zorbax Rx-C18 guard column (4.6 × 12.5 mm, 5 μm particle size) (Agilent Technologies, Waldbronn, Germany). Mobile phase A was 0.2% acetic acid (acetic acid:H_2_O; 0.2:99.8; *v*/*v*), and mobile phase B was 0.2% acetic acid and 80% methanol (acetic acid:MeOH:H_2_O; 0.2:80:19.8; *v*/*v*) and the solvent gradient profile as in Šola et al. [17]. The flow rate was 1 mL/min and the injected volume of the sample was 25 μL. Compounds were characterized according to their retention times and UV spectra compared with commercial standards. The wavelength at which the flavonoids were analyzed was 360 nm, for the phenolic acids analysis wavelength of 310 nm was used, and for *L*-ascorbic acid 254 nm. For the quantitative analyses, calibration curves were obtained by injecting known concentrations (in the range between 1 and 250 μg/mL) of the combined standard solution in triplicate.

### 3.6. Effect of Extracts on Antidiabetic Activity

Antidiabetic activity was determined as a function of enzymes α-amylase and α-glucosidase inhibition rate. The α-amylase inhibitory activity was tested as reported by Šola et al. [17]. In brief, equal volumes of extract and α-amylase were mixed, incubated 10 min at room temperature (RT), then the same volume of 1% aqueous starch was added and again the mixture was incubated for 10 min at RT. Finally, dinitrosalicylic acid was added and the mixture was incubated for 10 min at 100 °C. After cooling the mixture to RT, four time higher volume of water was added and the absorbance was measured at 544 nm using microplate reader Fluostar Optima (BMG Labtech GmbH, Offenburg, Germany). The percentage of α-amylase inhibition at a sample concentration of 0.8 mg/mL was calculated from the equation:

% inhibition = [100 − (A_t_ − A_tb_)/(A_c_ − A_cb_)] × 100, (1)

where A_t_ was the absorbance of the test (with amylase), A_tb_ was the absorbance of test blank (without amylase), A_c_ was the absorbance of control (with amylase), and A_cb_ was the absorbance of control blank (without amylase). Maltose was used as a positive control.

The inhibition of α-glucosidase was measured using the pre-incubation method as described by Salahuddin et al. [92] with slight modifications. In brief, extract (20 μL) was mixed with 100 μL *p*-nitrophenyl-α-D-glucopyranoside and pre-incubated for 10 min at 37 °C. The volume of 100 μL of α-glucosidase was added and re-incubated for 20 min at 37 °C. The enzyme reaction activity was terminated by the addition of 500 μL Na_2_CO_3_. The absorbance was measured at 405 nm using microplate reader Fluostar Optima. Enzyme inhibitory activity at a sample concentration of 0.55 mg/mL was calculated same as for the α-amylase inhibition. Acarbose was used as a positive control.

The antiglycation activity of extracts was determined according to Spínola et al. [91]. Briefly, bovine serum albumine solution (10 mg mL^−1^) was mixed with 0.1 M phosphate buffer, glucose (0.5 M) and sample extract. Control contained 70% ethanol instead of extract. Plates were incubated for 24 h at 37 °C and were analyzed at an excitation wavelength of 355 nm and emission wavelength of 460 nm. The antiglycation activity was expressed as percentage of BSA glycation inhibition at a sample concentration of 30.0 mg/mL and was calculated from the equation:

% inhibition = [(A_con_ − A_sample_)/A_con_] × 100, (2)

where A_con_ was the absorbance of control and A_sample_ the absorbance of sample.

### 3.7. Statistical Analysis

The data obtained were statistically processed in the Statistica 13.1 program (Stat Soft Inc., Krakow, Poland). All the experiments were performed in triplicate. The comparison of the sample means was carried out using one-way variance analysis (ANOVA) and Duncan’s New Multiple Range Test (DNMRT). Statistically significant values were those that differed at the *p* ≤ 0.05 level. Principal component analysis (PCA) and hierarchical clustering (HC) were performed to evaluate how close the samples were according to given parameters. Pearson’s correlation coefficients between the phytochemical content and bioactivities of microgreens were calculated.

## 4. Conclusions

Analyses revealed that garden cress had the lowest amount of soluble sugars. The highest amount of glucosinolates was found in Savoy cabbage and Brussels sprouts. The most potent inhibition of protein glycation was found in Brussels sprouts. Radish microgreens had the highest concentration of kaempferol both before and after digestion. A very high positive correlation (*r* = 0.830) between antiglycation potential and conjugated sinapic acid was recorded. All pre-digestion microgreens’ extracts showed antioxidant potential similar to or even higher than the Trolox standard. Kohlrabi’s antioxidant capacity was not changed after any phase of digestion. The extracts of all microgreens before digestion showed the same or higher degree of α-amylase inhibition than the standard antidiabetic drug acarbose. Pre-digestion radish, kale, Brussels sprouts and kohlrabi microgreen extracts inhibited α-glucosidase activity more strongly than acarbose. For the first time, microgreen antidiabetic potential after in vitro gastrointestinal digestion was analyzed, with kohlrabi microgreens showing the highest potential to inhibit the activity of α-amylase both in the initial phase of digestion and after intestinal digestion. The activity of α-glucosidase after intestinal digestion was also best inhibited by kohlrabi. Kohlrabi, Savoy cabbage, and garden cress microgreens retained their ability to inhibit α-amylase and α-glucosidase after digestion. The decreasing trend in Euclidean distance, indicating that the samples become more similar during digestion, suggests a homogenization of the measured parameters, that is phytochemicals’ concentration, antioxidant capacity, and antidiabetic potential, as digestion progresses. Overall, this trend implies that although the raw samples differ in their phytochemical profiles and bioactivities, digestion processes may diminish these variations, producing a more uniform response in terms of antioxidant and antidiabetic activities. Further, more detailed analyses of different types of derivatized phytochemicals, as well as screening after in vivo digestion, are highly recommended.

## Figures and Tables

**Table 1:** Amount, in mg/g dry mass (dm), of total phenolics (TP), total hydroxycinnamic acids (THCA), total flavonols (TFlo) and total tannins (TT) in Brassicaceae microgreens.

	Kohlrabi	Savoy Cabbage	Brussels Sprouts	Cauliflower	Radish	Garden Cress
TP (mg GAE/g dm)	23.30 ± 2.44 ^a^	18.60 ± 1.56 ^cd^	18.24 ± 1.75 ^cd^	19.92 ± 1.75 ^bc^	17.24 ± 2.76 ^d^	21.25 ± 1.52 ^b^
THCA (mg CinE/g dm)	24.54 ± 1.42 ^b^	30.54 ± 1.83 ^a^	22.68 ± 1.06 ^b^	24.01 ± 1.52 ^b^	22.93 ± 1.15 ^b^	24.64 ± 1.05 ^b^
TFlo (mg QE/g dm)	45.82 ± 2.14 ^c^	63.42 ± 3.52 ^a^	47.35 ± 2.36 ^c^	45.61 ± 2.12 ^c^	42.47 ± 2.51 ^c^	54.28 ± 3.85 ^b^
TT (mg TAE/g dm)	3.96 ± 0.29 ^a^	2.60 ± 0.20 ^cd^	2.32 ± 0.23 ^d^	3.29 ± 0.24 ^abc^	2.74 ± 0.21 ^bcd^	3.46 ± 0.15 ^ab^

Values represent mean ± standard deviation of three biological and three technical replicates (*n* = 9). Different letters indicate a significant difference among the values in a row (ANOVA, Duncan test, *p* ≤ 0.05). GAE = gallic acid equivalent, CinE = cinnamic acid equivalent, QE = quercetin equivalent, TAE = tannic acid equivalent.

**Table 2:** Concentration, expressed in mg/kg dry mass (dm), of vitamin C and individual phenolic compounds in Brassicaceae microgreens.

	Kohlrabi	Savoy Cabbage	Brussels Sprouts	Cauliflower	Radish	Garden Cress
before hydrolysis						
L-ascorbic acid	1495.05 ± 16.57 ^c^	1494.24 ± 9.00 ^c^	1489.16 ± 37.20 ^c^	1782.85 ± 40.93 ^b^	1956.43 ± 71.47 ^a^	1102.43 ± 46.32 ^d^
Ferulic acid	90.43 ± 2.59 ^a^	44.85 ± 0.90 ^b^	41.78 ± 0.22 ^c^	37.75 ± 0.71 ^d^	43.49 ± 0.52 ^bc^	37.45 ± 0.25 ^d^
Sinapic acid	132.68 ± 3.36 ^d^	149.09 ± 2.01 ^c^	108.19 ± 1.83 ^e^	74.54 ± 1.88 ^a^	125.30 ± 6.26 ^d^	169.13 ± 3.27 ^b^
Quercetin	nd	nd	nd	75.22 ± 2.63 ^b^	302.98 ± 7.64 ^a^	nd
Kaempferol	nd	nd	nd	nd	nd	nd
TIPA	223.11 ± 3.00 ^a^	193.94 ± 1.56 ^c^	149.97 ± 1.30 ^e^	112.29 ± 1.42 ^f^	168.79 ± 4.44 ^d^	206.59 ± 2.32 ^b^
TIF	nd	nd	nd	75.22 ± 1.86 ^b^	302.98 ± 5.40 ^a^	nd
TIP	223.11 ± 2.12 ^b^	193.94 ± 1.10 ^d^	149.97 ± 0.92 ^e^	187.51 ± 1.66 ^d^	471.77 ± 4.95 ^a^	206.59 ± 1.64 ^c^
TIC	1718.16 ± 7.65 ^c^	1688.18 ± 4.14 ^c^	1639.13 ± 16.66 ^c^	1970.36 ± 18.36 ^b^	2428.21 ± 32.27 ^a^	1309.02 ± 20.76 ^d^
after hydrolysis						
L-ascorbic acid	1029.15 ± 17.74 ^b^	852.83 ± 11.07 ^d^	947.17 ± 2.65 ^c^	1042.64 ± 8.78 ^b^	1067.98 ± 50.22 ^b^	1218.41 ± 45.95 ^a^
Ferulic acid	71.35 ± 2.77 ^d^	53.93 ± 1.60 ^e^	104.59 ± 0.39 ^a^	71.40 ± 4.55 ^d^	91.68 ± 5.70 ^b^	82.55 ± 2.87 ^c^
Sinapic acid	1486.98 ± 19.41 ^b^	1668.71 ± 29.46 ^a^	1335.13 ± 6.46 ^cd^	1283.15 ± 36.33 ^d^	1399.49 ± 79.60 ^bc^	889.07 ± 56.03 ^e^
Quercetin	416.88 ± 21.38 ^c^	274.21 ± 11.68 ^d^	476.11 ± 16.99 ^b^	638.66 ± 16.19 ^a^	101.25 ± 1.28 ^e^	667.12 ± 18.53 ^a^
Kaempferol	232.33 ± 11.28 ^d^	220.30 ± 13.80 ^d^	164.08 ± 3.98 ^e^	585.01 ± 31.20 ^c^	840.45 ± 34.08 ^a^	708.75 ± 23.87 ^b^
TIPA	1558.33 ± 21.85 ^b^	1722.64 ± 30.57 ^a^	1439.72 ± 6.07 ^cd^	1354.55 ± 40.81 ^d^	1491.17 ± 85.07 ^bc^	971.62 ± 58.13 ^e^
TIF	649.21 ± 32.35 ^d^	494.51 ± 25.31 ^e^	640.19 ± 20.81 ^d^	1223.67 ± 46.90 ^b^	941.71 ± 35.28 ^c^	1375.86 ± 37.70 ^a^
TIP	2207.54 ± 54.20 ^cd^	2217.14 ± 50.43 ^cd^	2079.91 ± 25.41 ^d^	2578.22 ± 86.28 ^a^	2432.87 ± 91.40 ^ab^	2347.48 ± 94.53 ^bc^
TIC	3236.68 ± 65.30 ^b^	3069.98 ± 56.92 ^b^	3027.08 ± 25.34 ^b^	3620.87 ± 90.46 ^a^	3500.85 ± 130.19 ^a^	3565.89 ± 137.41 ^a^

Values represent mean ± standard deviation of three biological and three technical replicates (*n* = 9). Different letters indicate a significant difference among the values in a row (ANOVA, Duncan test, *p* ≤ 0.05). TIPA = total identified phenolic acids (ferulic acid + sinapic acid), TIF = total identified flavonoids (quercetin + kaempferol), TIP = total identified phenolic compounds (TIPA + TIF), TIC = (TIP + *L*-ascorbic acid).

**Table 3:** Comparison of antioxidant capacity, expressed in inhibition %, of Brassicaceae microgreens extracts.

	Kohlrabi	Savoy Cabbage	Brussel Sprouts	Cauliflower	Radish	Garden Cress
ABTS	79.60 ± 1.66 ^abc^	78.00 ± 1.98 ^bc^	76.99 ± 0.11 ^cd^	81.95 ± 1.33 ^a^	75.05 ± 0.78 ^d^	80.19 ± 1.16 ^ab^
FRAP	94.48 ± 0.20 ^a^	93.83 ± 0.17 ^ab^	93.50 ± 2.88 ^bc^	93.75 ± 0.20 ^ab^	92.87 ± 2.28 ^c^	92.80 ± 0.81 ^c^
DPPH	64.76 ± 0.51 ^a^	66.32 ± 0.22 ^a^	65.27 ± 0.86 ^a^	65.40 ± 0.11 ^a^	58.77 ± 0.77 ^b^	53.92 ± 1.73 ^c^

Values represent mean ± standard deviation of three biological and three technical replicates (*n* = 9). Different letters indicate a significant difference among the values in a row (ANOVA, Duncan test, *p* ≤ 0.05). ABTS = 2,2′-azino-bis(3-ethylbenzothiazoline-6-sulfonic) acid, FRAP = ferric ion reducing antioxidant power, DPPH = 2,2-diphenyl-1-picrylhydrazyl.

**Table 4:** Amount of total phenolics (TP) and flavonoids (TF) released from Brassicaceae microgreens after in vitro gastrointestinal digestion.

	Kohlrabi	Savoy Cabbage	Brussels Sprouts	Cauliflower	Radish	Garden Cress
Digestion phase	**TP (mg GAE/g dm)**					
Initial	15.54 ± 1.59 ^a, B^	15.53 ± 2.14 ^a, AB^	15.44 ± 1.03 ^a, B^	16.04 ± 0.05 ^a, B^	14.43 ± 2.14 ^a, B^	15.75 ± 0.86 ^a, B^
Salivary	21.20 ± 1.66 ^a, A^	17.92 ± 1.75 ^a, A^	18.00 ± 1.58 ^a, A^	19.95 ± 1.36 ^a, A^	20.24 ± 2.79 ^a, A^	19.70 ± 0.43 ^a, A^
Gastric	12.67 ± 0.66 ^a, C^	12.85 ± 1.79 ^a, B^	12.48 ± 0.29 ^a, C^	13.00 ± 1.89 ^a, C^	12.05 ± 0.91 ^a, B^	13.90 ± 0.59 ^a, BC^
Intestinal	13.92 ± 1.12 ^ab, BC^	15.75 ± 1.35 ^a, AB^	12.11 ± 1.90 ^b, C^	12.34 ± 0.73 ^b, C^	11.59 ± 0.86 ^b, B^	13.50 ± 1.74 ^ab, C^
	**TF (mg QE/g dm)**					
Initial	20.35 ± 0.61 ^a, A^	20.41 ± 1.31 ^a, B^	21.08 ± 1.79 ^a, B^	20.79 ± 0.09 ^a, B^	19.78 ± 0.43 ^a, B^	19.49 ± 0.12 ^a, B^
Salivary	22.33 ± 1.68 ^a, A^	21.36 ± 0.35 ^ab, AB^	20.73 ± 0.59 ^bc, B^	20.56 ± 0.45 ^bc, B^	19.83 ± 0.41 ^c, B^	19.62 ± 0.20 ^c, B^
Gastric	20.22 ± 1.12 ^b, A^	22.32 ± 0.57 ^a, AB^	20.63 ± 1.18 ^b, B^	22.91 ± 1.12 ^a, A^	20.63 ± 0.52 ^b, B^	19.86 ± 0.60 ^b, B^
Intestinal	21.52 ± 0.90 ^c, A^	22.46 ± 1.34 ^abc, A^	23.54 ± 1.23 ^abc, A^	24.70 ± 1.14 ^a, A^	22.28 ± 0.84 ^bc, A^	24.21 ± 1.71 ^ab, A^

Values represent mean ± standard deviation of three biological and three technical replicates (*n* = 9). Different small letters indicate a significant difference among the values in a row, and different capital letters indicate a significant difference among the values in a column (ANOVA, Duncan test, *p* ≤ 0.05). dm = dry mass, GAE = gallic acid equivalent, QE = quercetin equivalent, CinE = cinnamic acid equivalent.

**Table 5:** Amount of *L*-ascorbic acid and individual phenolic compounds released from Brassicaceae microgreens after in vitro gastrointestinal digestion.

	Kohlrabi	Savoy Cabbage	Brussels Sprouts	Cauliflower	Radish	Garden Cress
Digestion phase	**L-ascorbic acid (mg/kg dm)**					
Initial	611.52 ± 45.36 ^bc, B^	535.73 ± 12.24 ^d, B^	564.85 ± 21.27 ^cd, A^	619.64 ± 28.06 ^bc, A^	671.84 ± 28.15 ^b, B^	1291.91 ± 31.24 ^a, A^
Salivary	593.27 ± 25.85 ^ab, B^	599.32 ± 53.42 ^ab, AB^	558.25 ± 38.72 ^b, A^	599.57 ± 41.05 ^ab, A^	673.33 ± 59.56 ^a, B^	592.76 ± 35.51 ^ab, C^
Gastric	690.78 ± 27.25 ^b, A^	657.23 ± 19.69 ^bc, A^	589.19 ± 16.37 ^d, A^	639.64 ± 25.15 ^c, A^	788.14 ± 13.75 ^a, A^	669.81 ± 15.27 ^bc, B^
	**Ferulic acid (mg/kg dm)**					
Initial	86.88 ± 2.04 ^e, D^	69.34 ± 3.16 ^f, C^	130.10 ± 2.06 ^b, C^	95.36 ± 0.99 ^d, C^	140.63 ± 1.84 ^a, B^	104.75 ± 1.83 ^c, B^
Salivary	102.80 ± 1.60 ^d, C^	95.00 ± 1.07 ^e, A^	149.64 ± 1.57 ^a, A^	105.36 ± 1.67 ^d, B^	141.38 ± 1.63 ^b, B^	118.87 ± 1.79 ^c, A^
Gastric	135.90 ± 1.73 ^b, A^	97.25 ± 1.91 ^d, A^	144.45 ± 1.55 ^a, B^	116.83 ± 2.55 ^c, A^	148.62 ± 1.87 ^a, A^	118.46 ± 3.03 ^c, A^
Intestinal	113.39 ± 2.05 ^c, B^	82.90 ± 1.39 ^e, B^	148.49 ± 1.99 ^a, AB^	103.23 ± 2.18 ^d, B^	132.65 ± 1.49 ^b, C^	104.09 ± 1.46 ^d, B^
	**Sinapic acid (mg/kg dm)**					
Initial	1802.65 ± 43.49 ^bc, C^	2262.30 ± 43.72 ^a, B^	1625.77 ± 96.88 ^c, A^	1632.05 ± 96.20 ^c, AB^	1949.65 ± 157.67 ^b, A^	1275.71 ± 47.94 ^d, B^
Salivary	1903.27 ± 47.87 ^b, B^	2396.71 ± 57.92 ^a, A^	1625.37 ± 45.77 ^c, A^	1457.42 ± 130.27 ^cd, B^	1960.97 ± 110.39 ^b, A^	1393.62 ± 99.48 ^d, B^
Gastric	2141.89 ± 29 47 ^b, A^	2296.71 ± 4.41 ^a, B^	1673.47 ± 61.99 ^c, A^	1674.92 ± 21.22 ^c, A^	2061.73 ± 57.98 ^b, A^	1567.88 ± 27.41 ^d, A^
Intestinal	1796.09 ± 48.38 ^b, C^	2197.03 ± 43.06 ^a, B^	1629.76 ± 37.62 ^c, A^	1564.89 ± 52.31 ^c, AB^	1872.74 ± 53.52 ^b, A^	1312.30 ± 48.64 ^d, B^
	**Quercetin (mg/kg dm)**					
Initial	240,88 ± 6.49 ^d, C^	213.78 ± 3.49 ^e, C^	318.24 ± 3.55 ^c, D^	487.62 ± 6.14 ^b, C^	111.51 ± 1.93 ^f, A^	530.02 ± 11.37 ^a, B^
Salivary	354.15 ± 8.87 ^d, B^	242.35 ± 9.93 ^e, B^	375.65 ± 4.68 ^c, B^	594.97 ± 9.82 ^a, B^	98.12 ± 1.59 ^f, B^	537.19 ± 10.05 ^b, B^
Gastric	432.49 ± 9.92 ^b, A^	267.35 ± 7.62 ^d, A^	408.26 ± 5.69 ^c, A^	613.58 ± 6.95 ^a, A^	108.21 ± 2.93 ^e, A^	608.81 ± 10.16 ^a, A^
Intestinal	189.99 ± 4.36 ^e, D^	241.87 ± 3.91 ^d, B^	356.69 ± 4.87 ^b, C^	466.60 ± 7.85 ^a, D^	112.26 ± 2.24 ^f, A^	315.49 ± 3.55 ^c, C^
	**Kaempferol (mg/kg dm)**					
Initial	121.43 ± 2.11 ^e, C^	155.31 ± 1.60 ^d, C^	114.60 ± 4.51 ^e, B^	434.95 ± 15.91 ^c, B^	662.05 ± 1.64 ^a, C^	515.13 ± 3.50 ^b, C^
Salivary	177.47 ± 2.14 ^e, B^	191.65 ± 4.04 ^d, AB^	135.24 ± 3.58 ^f, A^	491.01 ± 4.79 ^c, A^	698.74 ± 5.09 ^a, B^	542.34 ± 4.94 ^b, B^
Gastric	220.69 ± 5.47 ^d, A^	193.49 ± 6.77 ^e, A^	134.76 ± 3.64 ^f, A^	499.47 ± 9.77 ^c, A^	733.28 ± 5.96 ^a, A^	628.85 ± 9.70 ^b, A^
Intestinal	105.70 ± 5.23 ^e, D^	176.03 ± 11.76 ^d, B^	124.21 ± 4.89 ^e, B^	327.10 ± 20.03 ^b, C^	454.12 ± 19.25 ^a, D^	218.28 ± 11.37 ^c, D^
	**TIPA (g/kg dm)**					
Initial	1.89 ± 0.04 ^c, C^	2.33 ± 0.04 ^a, BC^	1.76 ± 0.09 ^c, A^	1.73 ± 0.10 ^c, AB^	2.09 ± 0.16 ^b, A^	1.38 ± 0.05 ^d, B^
Salivary	2.01 ± 0.05 ^b, B^	2.49 ± 0.06 ^a, A^	1.78 ± 0.05 ^c, A^	1.56 ± 0.13 ^d, B^	2.10 ± 0.11 ^b, A^	1.51 ± 0.10 ^d, B^
Gastric	2.28 ± 0.03 ^b, A^	2.39 ± 0.01 ^a, B^	1.82 ± 0.06 ^c, A^	1.79 ± 0.02 ^c, A^	2.21 ± 0.06 ^b, A^	1.69 ± 0.03 ^d, A^
Intestinal	1.91 ± 0.05 ^b, BC^	2.28 ± 0.04 ^a, C^	1.78 ± 0.04 ^c, A^	1.67 ± 0.05 ^d, AB^	2.01 ± 0.05 ^b, A^	1.42 ± 0.05 ^e, B^
	**TIF (g/kg dm)**					
Initial	0.36 ± 0.00 ^e, C^	0.37 ± 0.00 ^e, C^	0.43 ± 0.01 ^d, D^	0.92 ± 0.02 ^b, B^	0.77 ± 0.00 ^c, C^	1.05 ± 0.01 ^a, C^
Salivary	0.53 ± 0.01 ^c, B^	0.43 ± 0.01 ^d, B^	0.51 ± 0.00 ^c, B^	1.09 ± 0.01 ^a, A^	0.80 ± 0.00 ^b, B^	1.08 ± 0.01 ^a, B^
Gastric	0.65 ± 0.01 ^d, A^	0.46 ± 0.01 ^f, A^	0.54 ± 0.01 ^e, A^	1.11 ± 0.02 ^b, A^	0.84 ± 0.00 ^c, A^	1.24 ± 0.02 ^a, A^
Intestinal	0.30 ± 0.01 ^f, D^	0.42 ± 0.01 ^e, B^	0.48 ± 0.00 ^d, C^	0.79 ± 0.01 ^a, C^	0.57 ± 0.02 ^b, D^	0.53 ± 0.01 ^c, D^
	**TIP (g/kg dm)**					
Initial	2.25 ± 0.04 ^cd, C^	2.70 ± 0.04 ^ab, B^	2.19 ± 0.09 ^d, B^	2.65 ± 0.08 ^b, B^	2.86 ± 0.15 ^a, A^	2.43 ± 0.04 ^c, C^
Salivary	2.54 ± 0.05 ^b, B^	2.93 ± 0.07 ^a, A^	2.29 ± 0.04 ^c, AB^	2.65 ± 0.12 ^b, B^	2.90 ± 0.11 ^a, A^	2.59 ± 0.11 ^b, B^
Gastric	2.93 ± 0.04 ^b, A^	2.85± 0.02 ^b, A^	2.36 ± 0.07 ^c, A^	2.90 ± 0.04 ^b, A^	3.05 ± 0.06 ^a, A^	2.92 ± 0.05 ^b, A^
Intestinal	2.21 ± 0.05 ^d, C^	2.70 ± 0.05 ^a, B^	2.26 ± 0.04 ^d, AB^	2.46 ± 0.04 ^c, C^	2.57 ± 0.06 ^b, B^	1.95 ± 0.05 ^e, D^
	**TIC (g/kg dm)**					
Initial	2.86 ± 0.08 ^c, C^	3.24 ± 0.05 ^b, B^	2.75 ± 0.09 ^c, B^	3.27 ± 0.10 ^b, B^	3.54 ± 0.17 ^a, B^	3.72 ± 0.06 ^a, A^
Salivary	3.13 ± 0.08 ^b, B^	3.53 ± 0.11 ^a, A^	2.84 ± 0.08 ^c, AB^	3.25 ± 0.15 ^b, B^	3.57 ± 0.16 ^a, AB^	3.18 ± 0.14 ^b, B^
Gastric	3.62 ± 0.04 ^b, A^	3.51 ± 0.03 ^c, A^	2.95 ± 0.05 ^d, A^	3.54 ± 0.02 ^bc, A^	3.84 ± 0.05 ^a, A^	3.59 ± 0.05 ^bc, A^
Intestinal	2.21 ± 0.05 ^d, D^	2.70 ± 0.05 ^a, C^	2.26 ± 0.04 ^d, C^	2.46 ± 0.04 ^c, C^	2.57 ± 0.06 ^b, C^	1.95 ± 0.05 ^e, C^

Values represent mean ± standard deviation of three biological and three technical replicates (*n* = 9). Different small letters indicate a significant difference among the values in a row, and different capital letters indicate a significant difference among the values in a column (ANOVA, Duncan test, *p* ≤ 0.05). dm = dry mass, nd = not detected, TIPA = total identified phenolic acids (ferulic acid + sinapic acid), TIF = total identified flavonoids (quercetin + kaempferol), TIP = total identified phenolic compounds (TIPA + TIF), TIC = (TIP + *L*-ascorbic acid).

**Table 6:** Antioxidant capacity, expressed in % of inhibition, of Brassicaceae microgreen extracts after in vitro gastrointestinal digestion.

	Kohlrabi	Savoy Cabbage	Brussels Sprouts	Cauliflower	Radish	Garden Cress
Digestion phase	**ABTS (% inhibition)**					
Initial	21.95 ± 1.48 ^ab, A^	21.36 ± 1.67 ^ab, A^	21.35 ± 1.12 ^ab, AB^	22.89 ± 1.65 ^a, B^	19.71 ± 0.28 ^b, A^	21.80 ± 0.28 ^a, A^
Salivary	25.53 ± 2.57 ^ab, A^	21.21 ± 0.87 ^bc, A^	22.18 ± 1.72 ^abc, A^	27.14 ± 2.43 ^a, A^	17.05 ± 2.62 ^c, AB^	25.67 ± 1.60 ^ab, A^
Gastric	24.43 ± 1.42 ^a, A^	13.70 ± 0.48 ^c, B^	18.15 ± 2.53 ^b, B^	14.79 ± 2.14 ^bc, C^	12.81 ± 0.02 ^c, B^	14.63 ± 1.74 ^bc, B^
Intestinal	21.66 ± 3.34 ^a, A^	10.68 ± 2.37 ^bc, B^	11.27 ± 0.64 ^bc, C^	15.29 ± 0.79 ^b, C^	11.40 ± 3.35 ^bc, B^	6.60 ± 3.31 ^c, C^
	**DPPH (% inhibition)**					
Initial	33.07 ± 1.40 ^a, A^	24.85 ± 3.55 ^bc, A^	24.44 ± 4.24 ^bc, A^	38.19 ± 4.93 ^a, A^	18.33 ± 2.28 ^c, B^	30.26 ± 0.61 ^ab, A^
Salivary	16.56 ± 0.08 ^c, B^	22.70 ± 1.07 ^b, AB^	13.59 ± 0.37 ^d, C^	28.91 ± 0.81 ^a, B^	9.46 ± 0.36 ^e, C^	13.62 ± 2.50 ^d, B^
Gastric	20.23 ± 1.71 ^ab, B^	16.67 ± 1.34 ^abc, C^	15.96 ± 2.76 ^abc, C^	18.65 ± 1.27 ^abc, C^	28.39 ± 3.75 ^a, A^	14.16 ± 1.89 ^c, B^
Intestinal	25.92 ± 2.61 ^a, A^	18.03 ± 1.17 ^b, BC^	19.06 ± 1.73 ^b, AB^	18.37 ± 0.49 ^b, C^	16.01 ± 1.94 ^b, B^	17.18 ± 1.68 ^b, B^
	**FRAP (% inhibition)**					
Initial	76.37 ± 0.72 ^a, A^	75.38 ± 3.03 ^a, A^	76.20 ± 2.88 ^a, A^	76.92 ± 3.11 ^a, A^	73.24 ± 2.28 ^a, A^	76.98 ± 0.81 ^a, A^
Salivary	73.25 ± 1.23 ^a, B^	67.05 ± 2.95 ^c, B^	70.23 ± 0.89 ^ab, B^	71.84 ± 0.61 ^a, B^	67.25 ± 2.37 ^bc, B^	71.70 ± 0.44 ^a, B^
Gastric	71.76 ± 0.43 ^a, C^	69.20 ± 0.54 ^bc, B^	70.12 ± 0.82 ^b, B^	71.39 ± 0.86 ^a, B^	66.18 ± 0.58 ^d, B^	68.61 ± 0.32 ^c, C^
Intestinal	72.48 ± 0.25 ^a, BC^	68.00 ± 1.31 ^b, B^	63.15 ± 0.49 ^d, C^	66.92 ± 1.48 ^bc, C^	60.66 ± 0.98 ^e, C^	65.25 ± 2.08 ^cd, D^

Values represent mean ± standard deviation of three biological and three technical replicates (*n* = 9). Different small letters indicate a significant difference among the values in a row, and different capital letters indicate a significant difference among the values in a column (ANOVA, Duncan test, *p* ≤ 0.05). ABTS = 2,2′-azino-bis(3-ethylbenzothiazoline-6-sulfonic acid), FRAP = ferric ion reducing antioxidant power, DPPH = 2,2-diphenyl-1-picrylhydrazyl.

**Table 7:** Effect of in vitro gastrointestinal digestion on the potential of Brassicaceae microgreens to inhibit enzymes α-amylase and α-glucosidase.

	Kohlrabi	Savoy Cabbage	Brussels Sprouts	Cauliflower	Radish	Garden Cress
Digestion phase	**α-amylase (% inhibition)**					
Initial	60.51 ± 3.65 ^a, A^	30.76 ± 1.76 ^c, B^	45.68 ± 2.90 ^b, A^	18.16 ± 1.96 ^d, B^	18.28 ± 1.86 ^d, B^	29.07 ± 3.36 ^c, A^
Gastric	22.41 ± 1.44 ^bc, B^	13.57 ± 5.71 ^c, C^	33.76 ± 2.48 ^ab, B^	27.30 ± 1.50 ^ab, AB^	37.88 ± 7.14 ^a, A^	36.13 ± 7.67 ^a, A^
Intestinal	62.96 ± 3.39 ^a, A^	48.17 ± 7.14 ^b, A^	23.56 ± 4.57 ^d, B^	41.64 ± 8.94 ^bc, A^	31.84 ± 6.56 ^cd, AB^	40.90 ± 3.20 ^bc, A^
	**α-glucosidase (% inhibition)**					
Initial	14.62 ± 0.85 ^b, B^	6.91 ± 0.54 ^d, A^	10.41 ± 0.49 ^c, A^	15.27 ± 0.48 ^b, A^	18.70 ± 0.62 ^a, A^	7.54 ± 0.33 ^d, A^
Gastric	8.84 ± 0.90 ^a, C^	5.04 ± 0.83 ^c, A^	6.22 ± 0.53 ^bc, B^	5.70 ± 0.93 ^bc, B^	4.68 ± 0.60 ^c, B^	7.37 ± 0.84 ^ab, A^
Intestinal	19.22 ± 0.08 ^a, A^	4.39 ± 1.34 ^cd, A^	2.88 ± 1.82 ^d, B^	6.99 ± 1.79 ^bc, B^	6.26 ± 0.86 ^bcd, B^	8.39 ± 2.30 ^b, A^

Values represent mean ± standard deviation of three biological and three technical replicates (*n* = 9). Different small letters indicate a significant difference among the values in a row, and different capital letters indicate a significant difference among the values in a column (ANOVA, Duncan test, *p* ≤ 0.05).

**Table 8:** Pearson’s correlation coefficients between measured variables in (A) undigested microgreens, and (B) microgreens after intestinal phase of digestion. TP = total phenolics, TFlo = total flavonols, TT = total tannins, SS = soluble sugars, Glucosinol = total glucosinolates, ABTS = antioxidant capacity measured by the method ABTS, FRAP = antioxidant capacity measured by the FRAP method, DPPH = antioxidant capacity measured by the DPPH method, Antiglic = antiglication potential, Vit C = vitamin C, Fer = ferulic acid, Sin = sinapic acid, Q = quercetin, K = kaempferol, TIPA = total identified phenolic acids, TIF = total identified flavonoids, TIP = total identified phenolics, TIC = total identified compounds, c = free compound, cc = conjugated compound.

Variable	TP	THCA	TFlo	TT	Glucosinol	TProt	SS	Vit C (fc)	Fer (fc)	Sin (fc)	Q (fc)	TIPA (fc)	TIF (fc)	TIP (fc)	TIC (fc)	Vit C (cc)	Fer (cc)	Sin (cc)	Q (cc)	K (cc)	TIPA (cc)	TIF (cc)	TIP (cc)	TIC (cc)	ABTS	DPPH	FRAP	Antiglic
TP	1.000																											
THCA	0.000	1.000																										
TFlo	−0.020	0.903	1.000																									
TT	0.920	−0.108	−0.205	1.000																								
Glucosinol	−0.844	0.162	0.107	−0.909	1.000																							
TProt	−0.536	0.459	0.396	−0.750	0.863	1.000																						
SS	−0.683	−0.071	−0.343	−0.553	0.745	0.543	1.000																					
Vit C (fc)	−0.518	−0.249	−0.561	−0.260	0.495	0.177	0.898	1.000																				
Fer (fc)	0.693	0.005	−0.213	0.625	−0.443	−0.105	−0.031	−0.044	1.000																			
Sin (fc)	0.223	0.429	0.587	0.141	−0.435	−0.166	−0.514	−0.667	0.117	1.000																		
Q (fc)	−0.556	−0.377	−0.543	−0.216	0.201	−0.191	0.653	0.784	−0.212	−0.214	1.000																	
TIPA (fc)	0.528	0.349	0.368	0.427	−0.573	−0.187	−0.431	−0.562	0.596	0.867	−0.279	1.000																
TIF (fc)	−0.556	−0.377	−0.543	−0.216	0.201	−0.191	0.653	0.784	−0.212	−0.214	1.000	−0.279	1.000															
TIP (fc)	−0.394	−0.270	−0.436	−0.075	0.009	−0.264	0.528	0.619	−0.013	0.080	0.942	0.058	0.942	1.000														
TIC (fc)	−0.525	−0.277	−0.572	−0.226	0.388	0.056	0.863	0.970	−0.038	−0.495	0.902	−0.419	0.902	0.792	1.000													
Vit C (cc)	0.378	−0.537	−0.357	0.553	−0.771	−0.952	−0.590	−0.283	−0.098	0.240	0.186	0.145	0.186	0.244	−0.145	1.000												
Fer (cc)	−0.328	−0.839	−0.611	−0.343	0.149	−0.098	0.125	0.079	−0.234	−0.189	0.295	−0.270	0.295	0.213	0.127	0.311	1.000											
Sin (cc)	−0.236	0.495	0.167	−0.285	0.557	0.735	0.706	0.488	0.387	−0.185	0.078	0.044	0.078	0.097	0.410	−0.884	−0.429	1.000										
Q (cc)	0.531	−0.184	0.050	0.412	−0.440	−0.409	−0.772	−0.582	−0.159	−0.134	−0.636	−0.188	−0.636	−0.727	−0.678	0.441	0.014	−0.679	1.000									
K (cc)	−0.200	−0.368	−0.342	0.147	−0.306	−0.699	−0.027	0.274	−0.430	0.047	0.713	−0.177	0.713	0.680	0.424	0.732	0.192	−0.550	−0.043	1.000								
TIPA (cc)	−0.266	0.451	0.129	−0.317	0.583	0.749	0.735	0.508	0.381	−0.203	0.101	0.027	0.101	0.114	0.430	−0.887	−0.371	0.998	−0.697	−0.552	1.000							
TIF (cc)	0.161	−0.414	−0.250	0.372	−0.519	−0.822	−0.494	−0.131	−0.448	−0.044	0.194	−0.260	0.194	0.111	−0.068	0.868	0.166	−0.865	0.577	0.792	−0.877	1.000						
TIP (cc)	−0.058	−0.181	−0.310	0.288	−0.200	−0.565	0.063	0.459	−0.347	−0.374	0.526	−0.476	0.526	0.381	0.475	0.462	−0.197	−0.297	0.153	0.784	−0.319	0.734	1.000					
TIC (cc)	0.139	−0.378	−0.383	0.459	−0.502	−0.839	−0.235	0.183	−0.285	−0.144	0.451	−0.260	0.451	0.378	0.259	0.791	0.011	−0.622	0.314	0.887	−0.639	0.917	0.908	1.000				
ABTS	0.677	0.097	0.115	0.650	−0.510	−0.423	−0.633	−0.360	0.076	−0.198	−0.559	−0.122	−0.559	−0.624	−0.473	0.296	−0.452	−0.362	0.844	−0.040	−0.404	0.483	0.383	0.404	1.000			
DPPH	−0.065	0.301	0.068	−0.219	0.570	0.732	0.491	0.343	0.287	−0.567	−0.291	−0.315	−0.291	−0.412	0.139	−0.864	−0.322	0.782	−0.130	−0.757	0.781	−0.699	−0.275	−0.598	0.079	1.000		
FRAP	0.520	0.296	0.042	0.364	−0.020	0.318	0.095	0.046	0.742	−0.274	−0.485	0.150	−0.485	−0.452	−0.105	−0.543	−0.502	0.624	0.043	−0.746	0.606	−0.584	−0.295	−0.460	0.351	0.799	1.000	
Antiglic	−0.320	0.086	−0.198	−0.364	0.702	0.709	0.793	0.664	0.220	−0.677	0.106	−0.437	0.106	−0.042	0.503	−0.825	−0.106	0.830	−0.410	−0.529	0.845	−0.683	−0.153	−0.495	−0.216	0.913	0.610	1.000

**Table 9:** Pearson’s correlation coefficients between measured variables in microgreens after intestinal phase of digestion. TP = total phenolics, TF = total flavonoids, ABTS = antioxidant capacity measured by the method ABTS, FRAP = antioxidant capacity measured by the FRAP method, DPPH = antioxidant capacity measured by the DPPH method, Fer = ferulic acid, Sin = sinapic acid, Q = quercetin, K = kaempferol, TIPA = total identified phenolic acids, TIF = total identified flavonoids, TIP = total identified phenolics, TIC = total identified compounds.

Variable	TP	TF	Fer	Sin	Q	K	TIPA	TIF	TIP	TIC	ABTS	DPPH	FRAP	Amylase	Glucosidase
TP	1.000														
TF	−0.316	1.000													
Fer	−0.793	−0.054	1.000												
Sin	0.492	−0.695	−0.269	1.000											
Q	−0.129	0.870	−0.109	−0.531	1.000										
K	−0.551	0.175	0.087	−0.010	−0.175	1.000									
TIPA	0.439	−0.712	−0.195	0.997	−0.549	−0.003	1.000								
TIF	−0.539	0.798	−0.013	−0.409	0.616	0.668	−0.417	1.000							
TIP	0.147	−0.285	−0.220	0.833	−0.219	0.406	0.831	0.159	1.000						
TIC	0.147	−0.285	−0.220	0.833	−0.219	0.406	0.831	0.159	1.000	1.000					
ABTS	−0.117	−0.410	0.138	0.082	−0.078	−0.192	0.094	−0.213	−0.028	−0.028	1.000				
DPPH	0.273	−0.506	−0.007	0.082	−0.118	−0.653	0.083	−0.611	−0.284	−0.284	0.840	1.000			
FRAP	0.656	−0.309	−0.558	0.170	0.063	−0.626	0.129	−0.453	−0.138	−0.138	0.611	0.827	1.000		
Amylase	0.651	−0.463	−0.642	0.253	−0.226	−0.361	0.207	−0.460	−0.056	−0.056	0.562	0.718	0.919	1.000	
Glucosidase	0.187	−0.489	−0.142	−0.077	−0.313	−0.296	−0.090	−0.474	−0.387	−0.387	0.767	0.843	0.739	0.823	1.000

## Data Availability

The data that support the findings of this study are available from the corresponding author, [I.Š.], upon request.

## Supplementary Materials

The following supporting information can be downloaded at: https://www.mdpi.com/article/10.3390/ijms252111831/s1.
