# Supplementary material for: Phytochemical Composition and Functional Properties of Brassicaceae Microgreens: Impact of In Vitro Digestion
Source: Int J Mol Sci. 2024 Nov 4;25(21):11831. doi: 10.3390/ijms252111831 (PMC11546364; doi:10.3390/ijms252111831)
Supplement: Supplementary file 1 [file ijms-25-11831-s001.zip › ijms-3264893-supplementary.pdf]

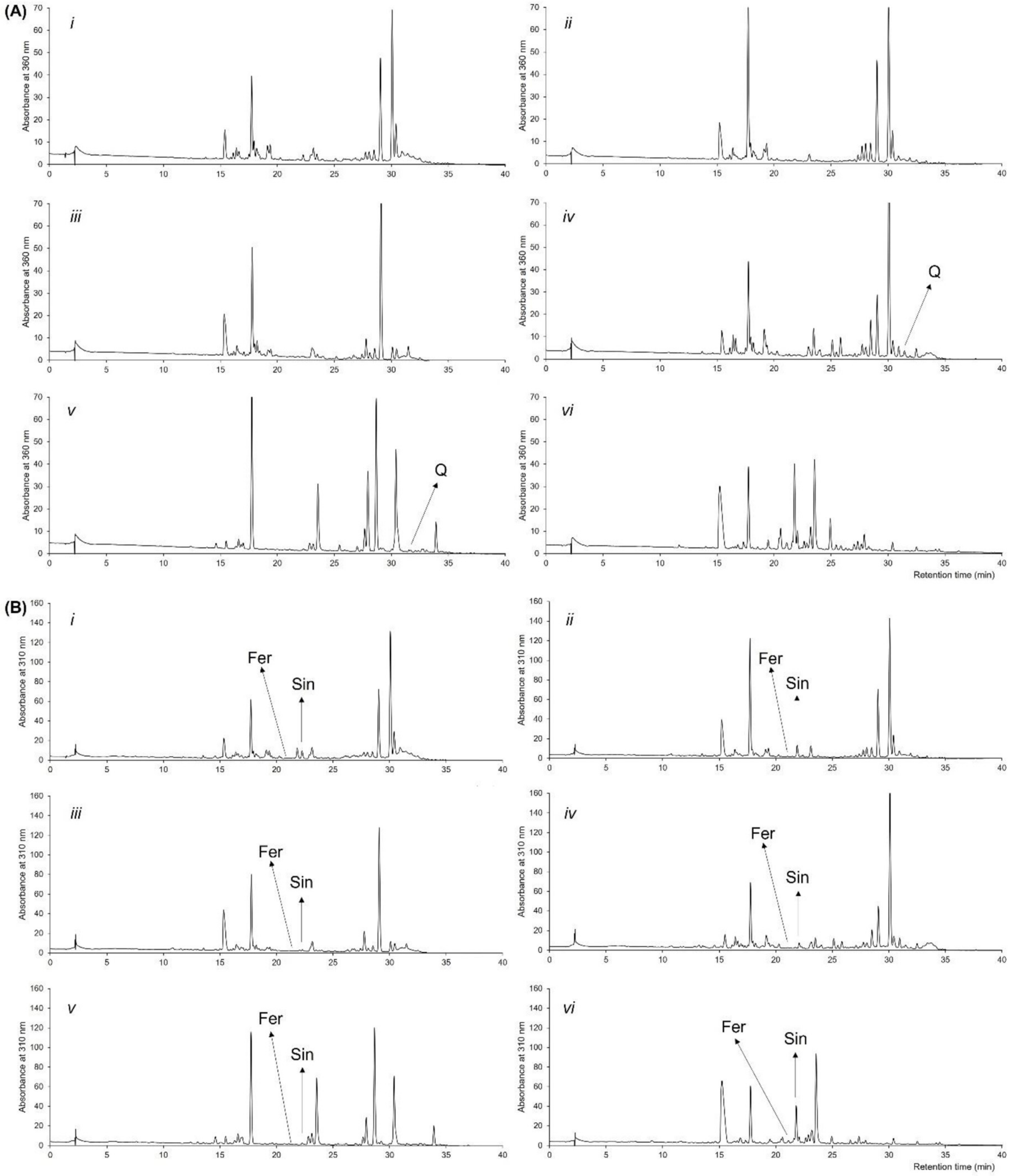

(A)
i
ii
iii
iv
v
vi
Q
Absorbance at 360 nm
Retention time (min)
(B)
Fer
Sin
Absorbance at 310 nm

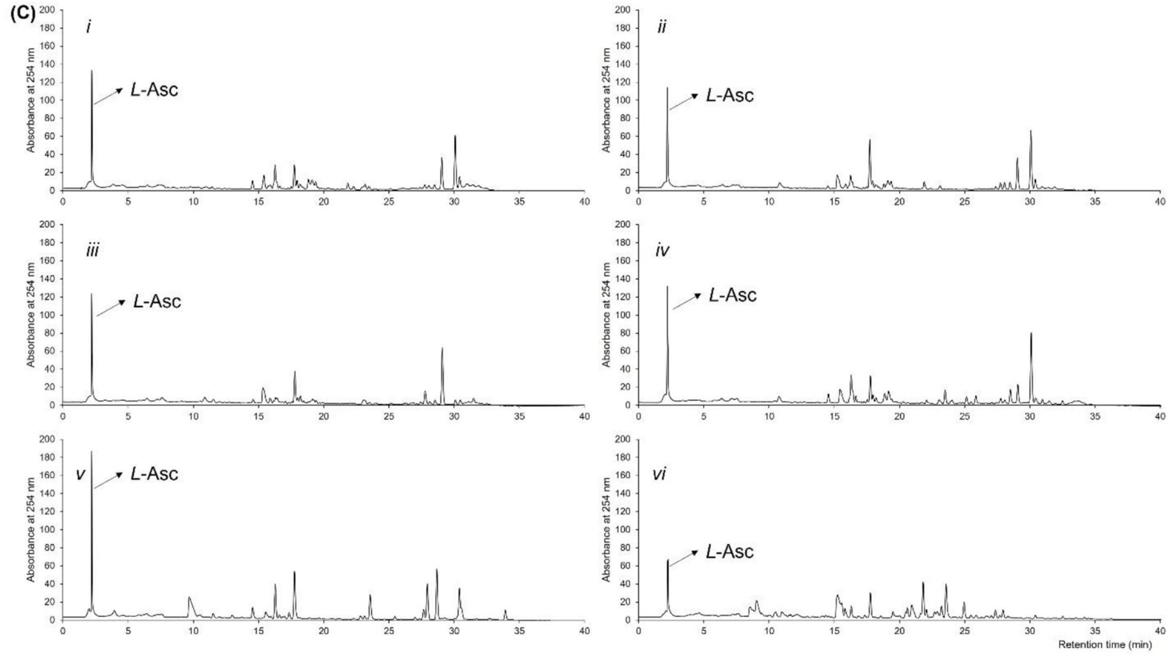

Figure S1. HPLC chromatograms of (*i*) kohlrabi (Brassica oleracea var. acephala gongylodes, sort Viola), (*ii*) kale (B. oleracea sabauda, sort Re d`inverno), (*iii*) Brussels sprouts (B. oleracea gemmifera, sort Bruxelles mezzo nano), (*iv*) cauliflower (B. oleracea botrytis, Palla di neve X), (*v*) radish (Raphanus sativus, Cherry belle) and (vi) garden cress (Lepidium sativum) extracts before hydrolysis recorded at (A) 360 nm, (B) 310 nm and (C) 254 nm.

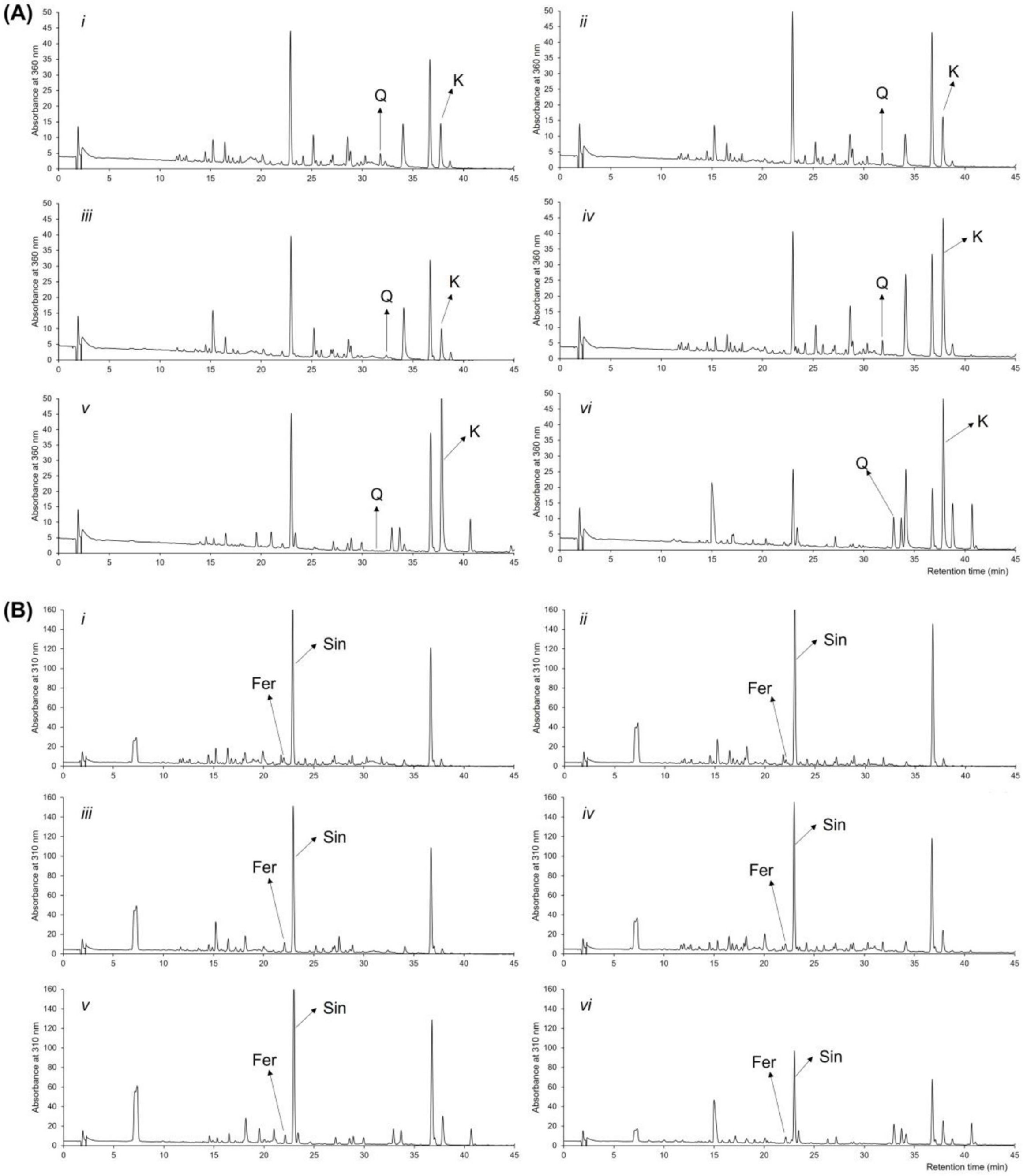
(A)
i
ii
iii
iv
v
vi
Absorbance at 360 nm
Q
K
Retention time (min)
(B)
Absorbance at 310 nm
Fer
Sin
Retention time (min)

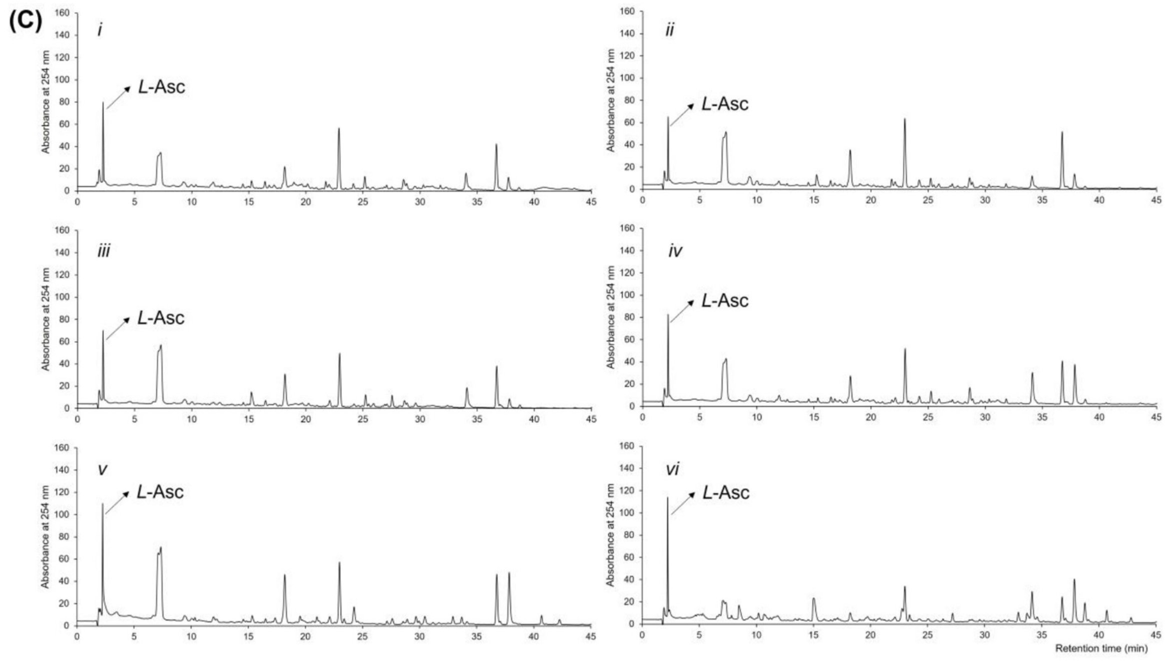

Figure S2. HPLC chromatograms of (*i*) kohlrabi (Brassica oleracea var. acephala gongylodes, sort Viola), (*ii*) kale (B. oleracea sabauda, sort Re d`inverno), (*iii*) Brussels sprouts (B. oleracea gemmifera, sort Bruxelles mezzo nano), (*iv*) cauliflower (B. oleracea botrytis, Palla di neve X), (*v*) radish (Raphanus sativus, Cherry belle) and (*vi*) garden cress (Lepidium sativum) extracts after hydrolysis recorded at (A) 360 nm, (B) 310 nm and (C) 254 nm;. Q = quercetin, K = kaempferol, Sin = sinapic acid, Fer = ferulic acid, L-Asc = L-ascorbic acid.
